# Cryptococcus neoformans Cda1 and Its Chitin Deacetylase Activity Are Required for Fungal Pathogenesis

**DOI:** 10.1128/mBio.02087-18

**Published:** 2018-11-20

**Authors:** Rajendra Upadhya, Lorina G. Baker, Woei C. Lam, Charles A. Specht, Maureen J. Donlin, Jennifer K. Lodge

**Affiliations:** a Department of Molecular Microbiology, Washington University School of Medicine, St. Louis, Missouri, USA; b Department of Medicine, University of Massachusetts Medical School, Worcester, Massachusetts, USA; c Edward A. Doisy Department of Biochemistry and Molecular Biology, Saint Louis University School of Medicine, St. Louis, Missouri, USA; University of British Columbia; Duke University Medical Center; University of Georgia

**Keywords:** chitin deacetylase, catalytic site, cell wall localization, chitosan, metal binding site, fungal virulence

## Abstract

Chitin is an essential component of the cell wall of Cryptococcus neoformans conferring structural rigidity and integrity under diverse environmental conditions. Chitin deacetylase genes encode the enyzmes (chitin deacetylases [Cdas]) that deacetylate chitin, converting it to chitosan. The functional role of chitosan in the fungal cell wall is not well defined, but it is an important virulence determinant of C. neoformans. Mutant strains deficient in chitosan are completely avirulent in a mouse pulmonary infection model. C. neoformans carries genes that encode three Cdas (Cda1, Cda2, and Cda3) that appear to be functionally redundant in cells grown under vegetative conditions. Here we report that C. neoformans Cda1 is the principal Cda responsible for fungal pathogenesis. Point mutations were introduced in the active site of Cda1 to generate strains in which the enzyme activity of Cda1 was abolished without perturbing either its stability or localization. When used to infect CBA/J mice, Cda1 mutant strains produced less chitosan and were attenuated for virulence. We further demonstrate that C. neoformans Cda genes are transcribed differently during a murine infection from what has been measured *in vitro*.

## Abstract (precis)

Cryptococcus neoformans is unique among fungal pathogens that cause disease in a mammalian host, as it secretes a polysaccharide capsule that hinders recognition by the host to facilitate its survival and proliferation. Even though it causes serious infections in immunocompromised hosts, reports of infection in hosts that are immunocompetent are on the rise. The cell wall of a fungal pathogen, its synthesis, composition, and pathways of remodelling are attractive therapeutic targets for the development of fungicides. Chitosan, a polysaccharide in the cell wall of C. neoformans is one such target, as it is critical for pathogenesis and absent in the host. The results we present shed light on the importance of one of the chitin deacetylases that synthesize chitosan during infection and further implicates chitosan as being a critical factor for the pathogenesis of C. neoformans.

## INTRODUCTION

*Cryptococcus* is a major fungal pathogen of immunocompromised patients and accounts for 15% of AIDS-related deaths annually (1). Due to the presence of a outer polysachharide capsule and a cell wall that is both complex and dynamic, it has been able to infect animal hosts and inhabit diverse environmental niches with a global presence (2–5). Two species, Cryptococcus neoformans and Cryptococcus gattii, are responsible for most clinical cases with C. neoformans affecting mainly immunocompromised individuals, while infections in immunocompetent people have been generally attributed to C. gattii (6, 7). There are exceptions, as C. gattii has been isolated from individuals infected with HIV, and certain genotypes of C. neoformans infect persons presumed to be immunocompetent (8, 9).

Chitin, a polymer made up of repeating units of β-1, 4 *N*-acetyl-D-glucosamine is an important component of the fungal cell wall matrix and is critical for maintaining cell wall integrity (10). Among the pathogenic fungi, C. neoformans is one of the few pathogens reported to efficiently deacetylate chitin to convert it to chitosan in the cell wall (11–13). The cell wall is essential for fungal survival. Since the majority of its components are unique to fungi and absent from the host, they present an attractive target for developing antifungal therapeutics. The cell wall is a dynamic organelle whose content and composition undergo constant modification in response to internal cellular homeostasis and external environmental cues, thereby enabling the fungal cells to survive under diverse stress conditions. However, this exclusive presence of cell wall components in a fungal pathogen makes them vulnerable to being sensed as pathogen-associated molecular patterns (PAMPs) by different pattern recognition receptors (PRRs) on host immune cells which in turn activate specific signaling pathways that orchestrate anticryptococcal defense mechanisms (14). To evade these host defense mechanisms, C. neoformans has evolved multiple resistance strategies such as shielding PAMPs from host recognition receptors, decoration of the surface or secretion of vesicles with molecules that skew host immune responses favoring fungal survival, mechanisms that counter host-induced oxidative and nitrosative stresses, and the ability to switch its morphology (15–21). While the encapsulation of the yeast with the polysaccharide capsule helps them to prevent host receptor engagement, shed capsular material also inhibits proinflammatory immune responses. By producing melanin, C. neoformans laccase stabilizes the cell wall and protects yeast cells from killing by microbicidal peptides, ingestion by macrophages, and killing by alveolar macrophages (22–24).

The chitin fiber cross-links glucans, thereby securing the integrity of the cell wall under different conditions of growth and during infection. However, the presence of chitin in the cell wall makes the yeast vulnerable to detection by the host immune system and degradation by host chitinases. Studies of mice and humans identified chitinases or chitinase-like proteins (C/CLP) belonging to a family of 18 glycosyl hydrolases that either degrade or bind to chitin polysaccharides. Some of these proteins include acidic mammalian chitinase, chitotriosidase, oviductin, and the chitinase-like proteins CHIL3, CHI3L1, CHI3L2, and CHIL4 (14). These data indicate that mammalian hosts have evolved robust immunologic defense strategies to deal with chitin-containing microbes. In addition to infecting mammals, *Cryptococcus* has also been reported to colonize various plant species where it encounters chitinase-mediated plant defense (25, 26). Furthermore, as *Cryptococcus* is a soil fungus, it is constantly exposed to chitinases from soil bacteria and amoeba where it has to protect its cell wall chitin from these microbial enzymes (27, 28). In Saccharomyces cerevisiae, chitosan is reported to be present only in the ascospore wall and is responsible for the increased resistance of the spores to environmental stress conditions (29–31). Microbial chitinases have been shown to be less effective at degrading deacetylated chitin or chitosan (32).

In C. neoformans, chitin synthase Chs3 and chitin synthase regulator Csr2 comprise the Chs/Csr system that produces the majority of vegetative cell wall chitin that is converted to chitosan. Three chitin deacetylases (Cdas) are expressed (Cda1, Cda2, and Cda3) with each contributing to chitosan synthesis. Strains of C. neoformans with either *CHS3*, *CSR2*, or all three chitin deacetylase genes (*CDA1*, *CDA2* and *CDA3*) deleted lack chitosan and have a common set of phenotypes, including sensitivity to a variety of cell wall stress conditions, and are rapidly cleared from infected mouse lungs (13). This indicates that chitosan is essential for the proper maintenance of cell wall integrity and virulence of C. neoformans, and Chs3, Csr2, and the Cdas are the principal proteins that catalyze its biosynthesis (11, 12).

Deletion of individual Cda genes did not affect the amount of cell wall chitosan when the yeast cells were grown in yeast extract-peptone-dextrose (YPD) medium, and they did not exhibit sensitivity to any of the cell wall stress conditions (12). Since we discovered that chitosan is critical for establishing infection in mammalian hosts, we wanted to investigate the role of individual Cda genes in chitosan biosynthesis during infection. The results presented here indicate that, of the three C. neoformans chitin deacetylases, Cda1 is the major contributor of fungal pathogenesis.

All three chitin deacetylases share similar protein sequence motifs for a cleavable N-terminal signal sequence and for C-terminal addition of a glycosylphosphatidylinositol (GPI) anchor. C. neoformans Cda2 (initially named MP98) was shown to be a potent stimulator of CD4 T cells (33). Recently, recombinant proteins of C. neoformans Cda1, Cda2, and Cda3 expressed in Escherichia coli were shown to induce protective immunity in mice against a C. neoformans infection when used as vaccines and delivered by glucan particles (34). These data indicate that C. neoformans Cda proteins have an inherent ability to modulate host immune responses. Therefore, we wanted to rule out the possibility that the absence of Cda1 protein in *cda1Δ* cells is responsible for its attenuated virulence. We also sought to distinguish whether the role of Cda1 in fungal pathogenesis depends on the presence of the protein as a component of the cell wall, its enzyme activity, or both of these features. To address this, we introduced loss-of-function point mutations in the catalytic residues of Cda1. Our findings revealed that Cda1 with its intact chitin deacetylase enzyme activity rather than its mere presence in the cell wall is necessary for virulence, implicating its role in chitin deacetylation as a critical factor for mediating fungal pathogenesis.

## RESULTS

### C. neoformans chitin deacetylases are functionally redundant under *in vitro* growth/stress conditions.

Previously we showed that deletion of three Cda genes was required to generate a chitosan-deficient strain of C. neoformans. This chitosan-deficient strain was found to be sensitive to growth when exposed to cell wall stressors and was avirulent in a murine model of infection (12, 13). This raised questions about the role each Cda had in chitosan biogenesis and virulence. We have already demonstrated that deletion of single Cda genes in C. neoformans did not affect the amount of cell wall chitosan when cultured in YPD at 30°C. Consistent with this, these strains did not display sensitivity to growth when exposed to either various cell wall stressors or to growth at 37°C. With a pulmonary infection, C. neoformans encounters multiple stresses such as different pH environments (pH 7 for the alveolar sac or pH 4 for the phagolysosome), limited nutrients, and exposure to reactive oxygen or nitrogen species. To assess the ability of the single Cda deletion strains to grow under these conditions, we measured their growth rate in a minimal medium of yeast nitrogen base (YNB) at two different pH values, pH 4 and pH 7, at 37°C. We found no difference in the rate of growth of each single deletion strain compared to their wild-type progenitor (data not shown). Similarly, growth rates of single Cda deletion and wild-type strains in RPMI 1640 medium containing 10% FBS at 37°C in the presence of 5% CO_2_ were similar (data not shown). Next, we spotted serial dilutions of yeast cultures on YNB agar (pH 4) in the presence of hydrogen peroxide (H_2_O_2_) (1 mM) or sodium nitrite (NaNO_2_) (0.75 mM). None of the single deletion strains had their growth altered by adding these compounds that generate reactive oxygen and nitrogen species, respectively. Overall, we could not find a phenotype for loss of a single Cda gene based on the ability of C. neoformans to grow under conditions that simulate some of the stresses they potentially encounter in a host.

### Deletion of *CDA1* severely attenuates C. neoformans virulence.

**FIG 1:**
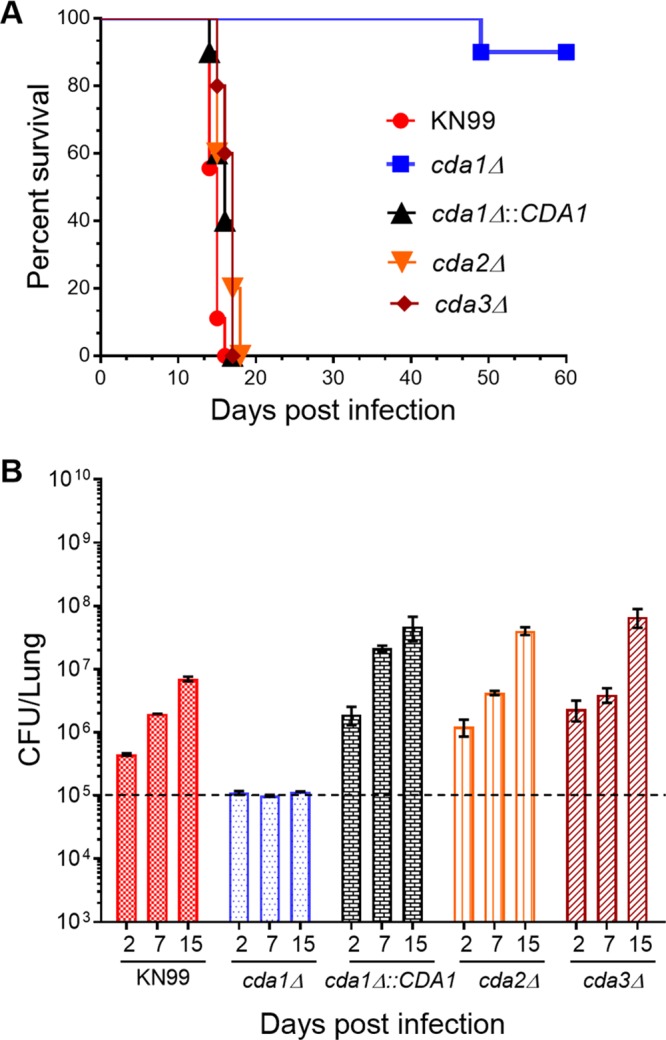
C. neoformans *cda1Δ* strain displays severely attenuated virulence. (A) CBA/J mice (4 to 6 weeks old, female) were infected intranasally with 10^5^ CFU of each strain. Survival of the animals was recorded as mortality of mice for 60 days postinfection. Mice that lost 20% of their body weight were considered ill and sacrificed. Data are representative of two independent experiments with 10 animals for each strain. Virulence was determined using Mantel-Cox curve comparison with statistical significance determined by the log rank test (*P < *0.0001 comparing KN99 with the *cda1*Δ strain). (B) Fungal burden in the lungs of mice infected with indicated strains at different days after infection. Data are the average of two experiments with four mice per group at each time point. The dashed line indicates the CFU of the initial infection for each strain.

Due to the critical importance of chitosan during infection, we wanted to determine whether its synthesis is dependent on any one of the three Cda genes during growth in the infected lung. To test this, we measured the virulence of each Cda deletion strain by intranasal infection of CBA/J mice. Deletion of *CDA1* rendered C. neoformans considerably less virulent than the wild-type KN99 strain (Fig. 1A). One of ten mice succumbed to infection, and the other nine mice looked very healthy throughout the length of the experiment without any sign of sickness (60 days postinfection [PI]). The deletion of either *CDA2* or *CDA3* did not impact virulence. Mice infected with either a *cda2Δ* or *cda3Δ* strain displayed comparable median survival times (17 days PI) to that of animals infected with wild-type KN99 (15 days PI). The importance of *CDA1* for fungal virulence is further supported by the observed restoration of virulence when the *cda1Δ* strain had a copy of the *CDA1* gene reintegrated at the endogenous *CDA1* locus. Infection with KN99, *cda2Δ*, *cda3Δ*, and *cda1Δ*::*CDA1* strains all showed significant fungal burdens ranging from 7.8 × 10^6^ to 3.3 × 10^8^ CFU/lung with a mean CFU/lung of 1.4 × 10^8^, 1.1 × 10^8^, 2.6 × 10^7^, and 7.7 × 10^7^, respectively, for animals infected with KN99, *cda2Δ*, *cda3Δ*, and *cda1Δ*::*CDA1* strains at 15 to 17 days PI (*n* = 10), when mice lost 20% of their preinoculation body weight, which was determined to establish the endpoint of the survival experiment. At these time points, fungal burdens in the brains ranged from 4 × 10^2^ to 7 × 10^4^ CFU/brain with a mean CFU/brain of 1 × 10^4^, 4 × 10^3^, 1.8 × 10^3^, and 1.7 × 10^4^, respectively, for animals infected with KN99, *cda2Δ*, *cda3Δ*, and *cda1Δ*::*CDA1* strains. In contrast, attenuated virulence of the *cda1Δ* strain was accompanied by a slow and incomplete clearance of the mutant yeast from the infected host. Mice were analyzed for fungal burden assays at 60 days PI, and there were variable numbers of CFU in lungs (ranging from 1 × 10^4^ to 5.8 × 10^5^ with a mean CFU/lung of 2.8 × 10^5^) and in the brain (ranging from 0 to 8.3 × 10^5^ with a mean CFU/brain of 8.7 × 10^4^). Some animals had begun to clear the infections, since the CFU/lung was around 10-fold less than the inoculum level.

We next wanted to determine whether there is a difference in the levels of fungal burden of different mutant strains in the lung soon after infection. To do this, we infected CBA/J mice with 10^5^ colony-forming units (CFU) of the wild-type strain and the individual Cda deletion strains. At days 2, 7, and 15 PI, lungs from the infected animals were subjected to fungal burden assays. As shown in Fig. 1B, KN99, *cda2Δ*, *cda3Δ*, and *cda1Δ*::*CDA1* strains all showed increased fungal burden days after infection. However, the fungal burden in the lungs of animals infected with the *cda1Δ* strain stayed constant and was similar to the initial inoculum. The attenuated virulence of the *cda1Δ* strain was apparent not only by reduced morbidity but also by the reduced fungal burden in the lungs during the first 2 weeks PI.

### C. neoformans Cda1 plays a major role in the synthesis of chitosan during infection.

**FIG 2:**
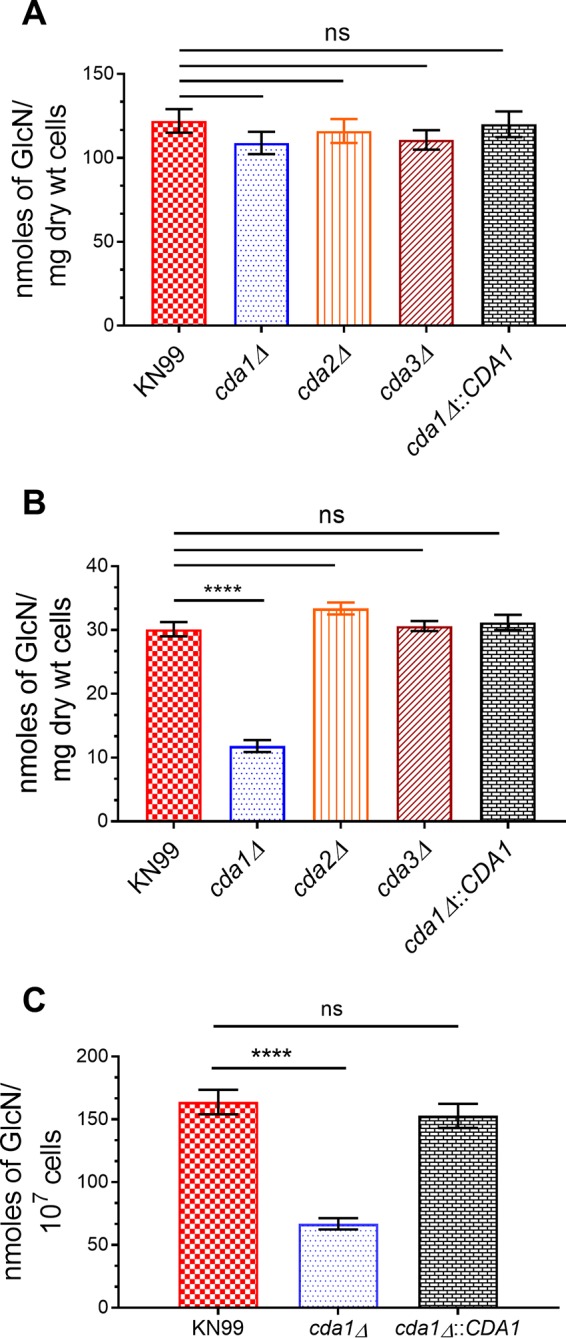
C. neoformans Cda1 plays a major role in the synthesis of chitosan under host infection conditions. (A) Chitosan levels of strains grown in YPD. The indicated strains were grown in YPD for 48 h. The amount of chitosan in the cell wall of the strains was quantified by the MBTH assay. Data are the averages for three biological experiments with two technical replicates and are expressed as nanomoles of glucosamine per milligram (dry weight) of yeast cells. (B) Chitosan levels of strains grown in RPMI containing 10% FBS and 5% CO_2_ at 37°C for 5 days. The indicated strains were grown in YPD for 48 h. Yeast cells were harvested, washed with PBS, and inoculated at 500 cells/μl in RPMI containing 10% FBS and incubated for 5 days at 37°C in the presence of 5% CO_2_. At the end of incubation, chitosan was measured by the MBTH assay and expressed as nanomoles of glucosamine per milligram (dry weight) of cells. Data represent the averages for three biological experiments with two technical replicates. (C) Chitosan levels of strains growing in the murine lung. Mice (CBA/J; four mice per group) were intranasally inoculated with 10^7^ CFU of each strain. On day 7 PI, lungs were excised and homogenized, and the lung tissue was removed by alkaline extraction, leaving the fungal cells to be harvested, counted, and subjected to the MBTH assay. Data were expressed as nanomoles of glucosamine per 10^7^ cells. Significant differences between the groups were compared by one-way ANOVA, followed by Bonferroni’s multiple-comparison test (*P < *0.0001 comparing KN99 with any other strain; ns, not significant).

The ability to synthesize chitosan is important for the integrity of the C. neoformans cell wall, since chitosan-deficient strains displayed sensitivity to growth under cell wall stress conditions (12). Individual Cda deletion strains did not exhibit either a cell wall defect or sensitivity to cell wall stress conditions when grown in YPD (12; data not shown). Therefore, we measured chitosan levels in wild-type, single Cda deletion strains, and the corresponding *CDA1* complemented strains using the 3-methyl-2-benzothiazolinone hydrazone hydrochloride hydrate (MBTH) method described in Materials and Methods. Strains were grown in YPD for 2 days, similar to the way inocula were grown for the virulence assay. We observed no significant differences in the cellular chitosan levels between the wild-type KN99 strain and the corresponding single Cda deletion strains (Fig. 2A). However, since the *cda1Δ* strain was the only strain that displayed attenuated virulence in the mouse survival experiment, we asked whether deletion of *CDA1* specifically affected the ability of the cells to synthesize chitosan either in the infected lung or when grown under conditions that mimic the host, such as RPMI 1640 medium containing 10% fetal bovine serum (FBS), 5% CO_2_, and 37°C. To test this hypothesis, we grew cells in YPD at 30°C for 48 h and then transferred the cells to RPMI medium containing 10% FBS at 37°C in the presence of 5% CO_2_ and grew the cells for 5 more days. After 5 days under host-mimicking conditions, the levels of chitosan in the *cda1Δ* cells were significantly reduced (2.5-fold less than that of KN99), whereas the *CDA1* reconstituted strain showed wild-type levels of chitosan (Fig. 2B). This difference in the amount of chitosan was not observed for either the *cda2*Δ and *cda3Δ* cells (Fig. 2B). Next, we tested whether chitosan production in the *cda1Δ* cells was affected when the cells were grown in infected lung. To do this, we inoculated mice with 10^7^ CFU of strain KN99 or the *cda1Δ* strain. At 7 days PI, we harvested the fungal cells from the infected lung and measured the cellular chitosan content. Again, as shown in Fig. 2C, unlike YPD growth conditions, but similar to the RPMI growth conditions, the levels of chitosan present in the *cda1Δ* strain were significantly reduced (2.4-fold less) compared to KN99 cells, whereas the strain reconstituted with *CDA1* showed wild-type levels (Fig. 2C). On the basis of these data, we concluded that Cda1 removes the majority of the acetyl groups from chitin in the generation of chitosan during host infection and that the inability to produce chitosan is likely responsible for the attenuated virulence of the *cda1Δ* strain.

### Specific mutation of conserved residues at the catalytic site abolishes chitin deacetylase activity of C. neoformans Cda1.

C. neoformans Cda genes were initially identified by the presence of a chitin/polysaccharide deacetylase domain in their putative protein products. Out of the four predicted deacetylase genes, two of them (Cda2/MP98 and Fpd1/d25) were previously shown to be highly immune reactive mannoprotein antigens (33, 35). Our previous genetic study involving the generation of a chitosan-deficient *cda1Δcda2Δcda3Δ* strain indicated that Fpd1 has an insignificant role in the deacetylation of cell wall chitin (12). All four proteins are predicted to contain a N-terminal signal sequence, and the three Cda proteins each have a C-terminal site for the addition of a glycosylphosphatidylinositol (GPI) anchor, which is not present for Fpd1 (12). Accordingly, Cda2 was demonstrated to be distributed in the cell wall, cell membrane, and secreted fractions. The presence of Cda2 in the cell membrane was found to be critical for its effective deacetylase activity (36). Therefore, we wanted to characterize the deacetylase enzyme activity of Cda1 and its distribution in the cell and also wanted to determine to what extent Cda1 as an immunogen or Cda1 as the enzyme contributes to the virulence of C. neoformans. To this end, we generated a strain of C. neoformans in which catalytic residues of Cda1 were mutated to abolish its chitin deacetylase activity, while otherwise causing no alteration to its stability and cellular localization.

**FIG 3:**
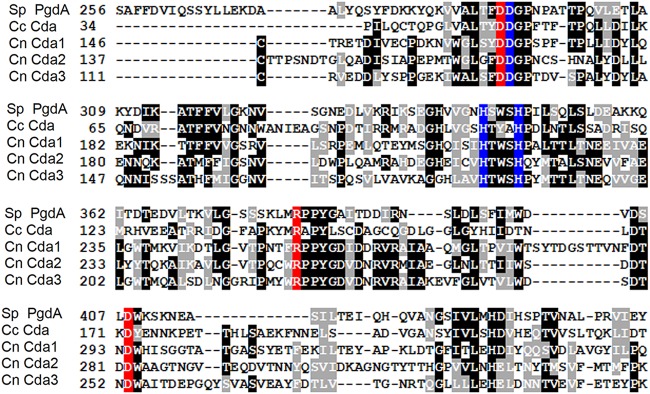
Identification of potential active site residues of C. neoformans Cda1 by sequence alignment. Multiple-sequence alignment of polysaccharide deacetylases. The sequences of C. neoformans Cdas (Cn Cda1, Cn Cda2, and Cn Cda3 [CNAG_05799, CNAG_01230, and CNAG_01239, respectively]) were aligned by the T-coffee program to the peptidoglycan *N*-deacetylase of *S. pneumoniae* (Sp PgdA) (GenBank accession number EDK82277.1) and Cda of *C. lindemuthianum* (Cc Cda) (GenBank accession number AAT68493.1) (60, 61). Identical amino acids are in black boxes, and similar amino acids are in gray boxes. The conserved residues corresponding to catalytic site and metal binding pocket are colored red and blue, respectively.

The mechanisms of deacetylase catalysis were first reported for the pathogen Streptococcus pneumoniae and the enzyme peptidoglycan GlcNAc deacetylase (SpPgdA). In this study, Blair and coworkers found that the Asp-275 residue coordinates acetate binding and the conservative substitution of it with asparagine completely inactivated the enzyme without adversely affecting protein stability (37). Additionally, they discovered a zinc binding triad in SpPgdA consisting of amino acids Asp-276, His-326, and His-330 and that mutation of His-326 to Ala also abolished enzyme activity (37). The same authors extended their investigation to determine the crystal structure and the mechanism of catalysis of a Cda from Colletotrichum clindemuthianum, a plant fungal pathogen (38). They demonstrated that Asp-49, Arg-142, and His-206 of *C. clindemuthianum* are directly involved in acetate binding and are critical for enzyme catalysis. They also identified Asp-167, His-216, and His-220 as the zinc binding triad important for deacetylase activity (38). We aligned our three chitin deacetylase protein sequences with the peptidoglycan deacetylases from *S*. *pneumoniae* and *C. clindemuthianum* and identified the three catalytic residues, D166, R254, and D294 in Cda1, as well as zinc-binding residues, D167, H216, and H220 in Cda1 (Fig. 3). We disrupted the acetate binding residues in Cda1: *cda1*^*D166N*,*R254A*,*D294N*^ (Cda1^CS^:Cda1 catalytic site mutant) and the zinc binding pocket, *cda1*^*D167N*,*H216A*,*H220A*^ (Cda1^MB^:Cda1 metal binding pocket mutant). The designs of the DNA constructs used for gene replacement at the *CDA1* locus are shown in Fig. S1 in the supplemental material. The *cda1*Δ and *cda2Δ3Δ* strains (12) were biolistically transformed with the constructs shown in Fig. S1. We confirmed each transformant by diagnostic PCR and Southern blotting (data not shown). The *cda1Δ* strain was created by replacing *CDA1* with the hygromycin (HYG) drug cassette. Thus, we identified successful transformants of the *cda1Δ* strain by the loss of the hygromycin (HYG)-resistant drug cassette when it was replaced with the mutated *CDA1* constructs (Fig. S1). In the case of the transformation of the *cda2Δ3Δ* strain with the *cda1*^CM^, *cda1*^MB^, and *CDA1* alleles, we initially screened for G418 resistance and then serially passaged positive transformants on nonselective media before screening them again on G418 drug plates. We then identified the transformants by diagnostic PCR at the 5′ junction of the integration event. The PCR-positive isolates showed a slow growth phenotype in YPD with small colonies similar to those of the *cda1Δ2Δ3Δ* strain, indicating that the mutant allele has replaced its wild-type gene at the desired *CDA1* locus and that the mutation of the predicted catalytic site may have abolished the chitin deacetylase activity (12).

10.1128/mBio.02087-18.1 FIG S1 Generation of C. neoformans *CDA1* gene fragment with site-specific point mutations in the catalytic and metal binding sites. (A) Fragments A and C were amplified by PCR using KN99 genomic DNA as the template. Fragment B (asterisks denote mutated residues) was generated initially by gene synthesis and amplified by PCR. Overlap PCR technology was used to assemble all three fragments to make a full-length genomic region of the *CDA1* gene. (B) Representative schematic of the homologous recombination of the wild-type *CDA1* and the two mutant constructs at the *CDA1* genomic locus when the *cda1Δ* strain was used as the recipient strain for biolistic transformation. C. neoformans *CDA1* gene is surrounded by the L33 gene (large subunit of ribosomal protein) and SRP (signal recognition particle) on the 5′ and 3′ side, respectively. *CDA1* gene constructs were made in such a way to insert the G418 drug selection marker between the *CDA1* and *L33* genes. The locations of the diagnostic primers used for the identification of the positive transformants are indicated. (C) Plasmid map and DNA sequence of the plasmid pCDA1WT (JL553). Download FIG S1, DOCX file, 0.3 MB. Copyright © 2018 Upadhya et al. 2018 Upadhya et al. This content is distributed under the terms of the Creative Commons Attribution 4.0 International license.

**FIG 4:**
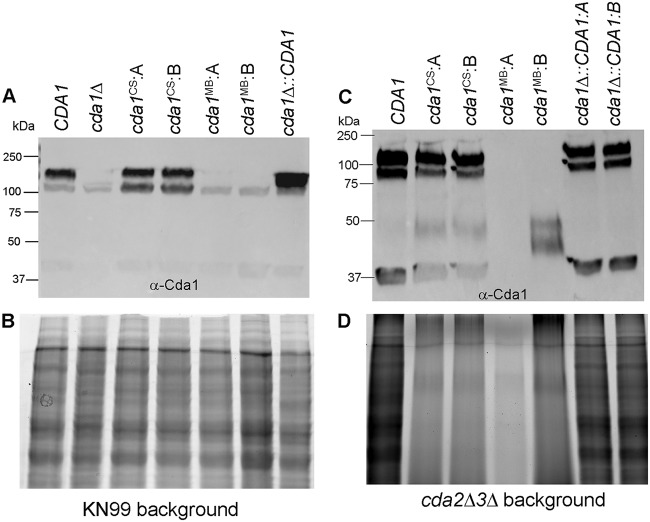
Effects of changes in amino acids to the catalytic site and metal binding site of Cda1 on protein stability. (A) Western blot analysis of the whole-cell lysates of the wild-type strain and the corresponding Cda1 mutant strains. Equal amounts of protein quantified by BCA protein assay were separated on a 12% Stain-Free Tris-glycine gels. Proteins were transferred to a nitrocellulose membrane and probed with a C. neoformans Cda1-specific polyclonal antibody. (B) Total proteins detected by the photo-activation of the stain-free gel used in the immunoblot shown in panel A. (C) Immunoblot analysis of the whole-cell lysates of catalytic and metal binding site mutants generated in *cda2Δ3Δ* background. (D) Visualization of total proteins present in each lane of the blot in panel C using stain-free technology.

We first wanted to verify that the specific point mutations engineered in the *CDA1* gene did not affect Cda1 protein expression and turnover. We measured the mutated Cda1 protein levels present in the KN99 or *cda2Δ3Δ* background by Western blot analysis using a C. neoformans Cda1-specific polyclonal antibody. We observed stable production of the mutated Cda1 protein as seen in the lysates of two independent isolates of the catalytic point mutants (Cda1^CS^: isolates A and B) generated in the KN99 background (using the *cda1Δ* strain as the recipient strain) (Fig. 4A, Cda1^CS^, isolates A and B). The absence of a Cda1-specific signal from the lysates of *cda1Δ* cells established the specificity of the polyclonal antibody (Fig. 4A). We were also able to engineer mutations at residues Asp-167, His-216, and His-220 to Asn, Ala, and Ala residues, respectively, to disrupt the predicted metal binding pocket. Two independent isolates (Cda1^MB^; isolates A and B) were used for immunoblot analysis. However, we did not see Cda1-specific signal, suggesting that disruption of the zinc binding pocket destabilized the protein and targeted it for degradation (Fig. 4A). Equal amount of the total protein loaded onto each lane as determined by the bicinchoninic acid (BCA) assay was verified by photo-activating the stain-free gel using stain free imaging technology (Fig. 4B) (Bio-Rad Laboratories, USA) (39). Similar results were obtained when the lysates of mutants generated in the *cda2Δ3Δ* background were subjected to Western blot analysis, as shown in Fig. 4C, and the total protein present in each lane was visualized by the activation of the stain-free gel (Fig. 4D).

**FIG 5:**
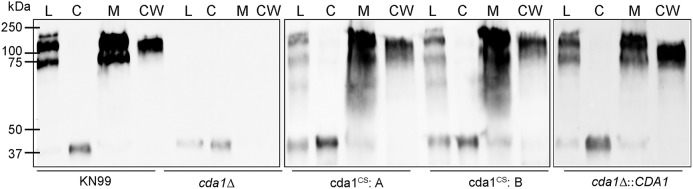
Disruption of C. neoformans Cda1 catalytic site did not interfere with its cellular localization. The indicated strains were streaked onto YPD agar and incubated for 4 days at 30°C. Cells were collected from the plates, washed with PBS, and subjected to subcellular fractionation. Equal quantities of protein as determined by the BCA method were separated on 10% SDS gel and analyzed by Western blotting using a C. neoformans Cda1-specific polyclonal antibody. Lanes: L, total cell lysate; C, cytosol fraction; M, cell membrane fraction; CW, cell wall fraction.

We have previously shown that Cda2 is localized in the plasma membrane, cell wall, and secreted fractions of C. neoformans and that localization of Cda2 in the cell membrane is essential for the synthesis of chitosan, whereas cell wall-localized Cda2 is not efficient at producing the polymer (36). We thus wanted to determine whether replacement of the catalytic residues in Cda1 affected its localization. We fractionated independently isolated strains of mutated Cda1 present in the KN99 background and subjected them to Western blot analysis with the Cda1-specific antibody. Mutation of the catalytic residues of the Cda1 protein (Cda1^CS^) had no measurable effect on its localization in the membrane fraction compared to its wild-type Cda1 counterpart as can be seen on lanes marked M for membrane and CW for cell wall fractions in Fig. 5. Thus, disruption of the catalytic residues of Cda1 did not impact its stability or its localization.

**FIG 6:**
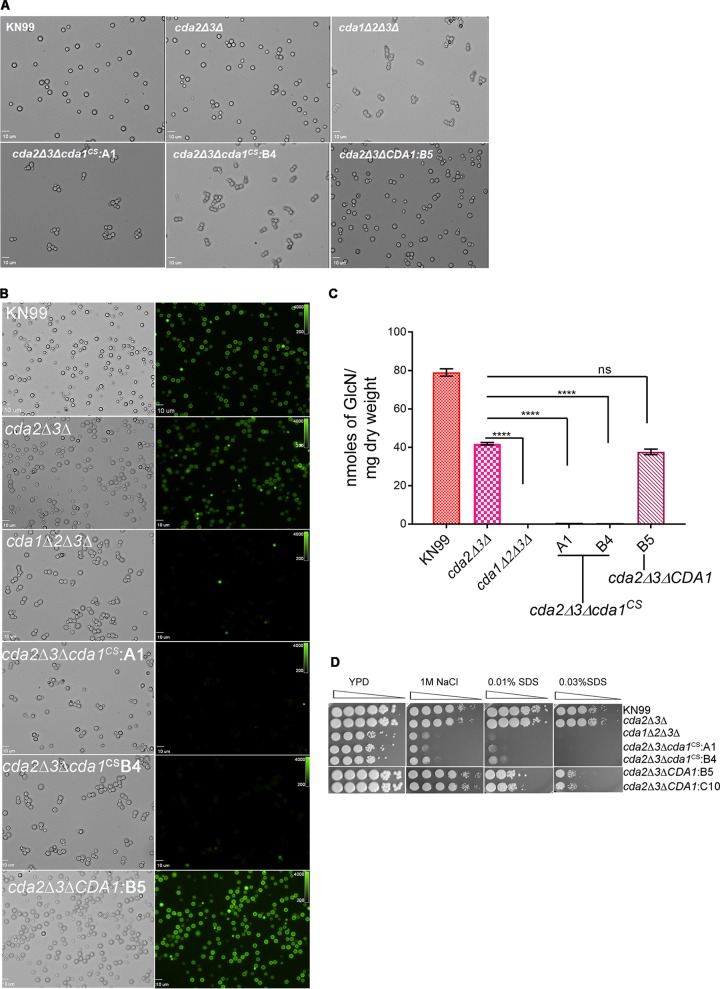
Point mutations in the catalytic site of Cda1 expressed in a *cda2Δ3Δ* strain results in chitosan deficiency and phenotypes similar to those of the chitosan-deficient *cda1Δ2Δ3Δ* strain. (A) Cda1 catalytic point mutant strains generated in the *cda2Δ3Δ* background display a budding defect when grown in YPD medium similar to the budding defect observed for a chitosan-deficient *cda1Δ2Δ3Δ* strain. The wild-type and mutant strains were grown in YPD for 36 h, collected, washed, and visualized with an Olympus BX61 microscope, and photographs were taken at 100× magnification. (B) The wild-type, corresponding Cda deletion, and mutant strains were grown as described above in panel A and stained with Eosin Y to detect cell wall chitosan. Bright-field and ﬂuorescence isothiocyanate panels are shown. Photographs were taken at 100× magnification. (C) Quantitative determination of cell wall chitosan in wild-type and various Cda deletion and mutant strains by the MBTH assay. Cells were grown in YPD for 48 h, collected, washed, and used for the assay. Data represent the averages for three biological and two technical replicates. Significant differences between the groups were compared by one-way ANOVA, followed by Dunnett’s multiple-comparison test (****, *P* < 0.0001 comparing the chitosan amount for each strain to that for the *cda2Δ3Δ* strain). (D) Wild-type and various C. neoformans Cda deletion and catalytic point mutant strains were grown in YPD for 36 h. Cells were harvested, washed, and diluted to an OD_650_ of 1.0. Four microliters of 10-fold serially diluted cell suspension was spotted on YPD agar alone and YPD agar containing cell wall stress reagents NaCl and SDS. The plates were incubated for 4 days at 30°C and photographed.

Previously we have shown that chitosan-deficient C. neoformans strains generated either by the deletion of *CHS3*, *CSR2*, or all three Cda genes (*CDA1*, *CDA2*, and *CDA3*) exhibited cell wall defects when plated on media that induced cell wall stress and did not stain with eosin Y, an anionic dye that binds preferentially to chitosan (11). Therefore, we reasoned that if the specific mutations created in the catalytic site of Cda1 interfere with its enzyme activity, then the mutant strain generated in the *cda2Δ3Δ* background (*cda2Δ3Δcda1*^CS^) should also exhibit phenotypes similar to those shown by chitosan-deficient strains. The chitosan-deficient strains show a budding defect in which daughter cells fail to separate from the mother cell, generating clumps of cells when grown in rich media (11, 12) (*cda1Δ2Δ3Δ* strain in Fig. 6A). We observed a similar budding defect for two independent isolates (A1 and B4) of the mutated Cda1 in the *cda2Δ3Δcda1^CS^* strain (Fig. 6A). This phenotype is consistent with each isolate bearing an enzymatically inactive Cda1. The control strain with wild-type CDA1 in a *cda2Δ3Δ* background exhibits no budding defect (isolate B5; Fig. 6A). Previously we demonstrated that the lack of Eosin Y staining of *cda1Δ2Δ3Δ* cells correlated with the absence of chitosan (12). The two independent isolates of the mutant strain (*cda2Δ3Δcda1^CS^* A1 and B4 isolates) also showed no binding of Eosin Y to the cell wall, similar to the *cda1Δ2Δ3Δ* strain (Fig. 6B). The binding of Eosin Y is restored in the *cda2Δ3Δ* strain in which wild-type Cda1 is being expressed (*cda2Δ3ΔCDA1*:B5 strain in Fig. 6B). We further confirmed the chitosan deficiency of these mutant strains by the chemical determination of cell wall-associated chitosan using the MBTH assay (Fig. 6C). Earlier studies have shown that the absence of chitosan in the cell wall rendered cells sensitive to cell wall stress reagents (12). We tested two independent isolates of *cda2Δ3Δcda1*^CS^ with the cell wall stressors 1 M NaCl and 0.01% or 0.03% SDS and observed phenotypes similar to those of the *cda1Δ2Δ3Δ* strain (Fig. 6D). We did not observe the cell wall stress phenotypes in the control *cda2Δ3ΔCDA1* strains. Thus, we conclude that site-specific mutation of the conserved catalytic residues of Cda1 abolishes its chitin deacetylase activity while retaining its protein stability and cellular location, even in the absence of the *CDA2* and *CDA3* genes.

### Activity of Cda1 is primarily responsible for fungal pathogenesis.

**FIG 7:**
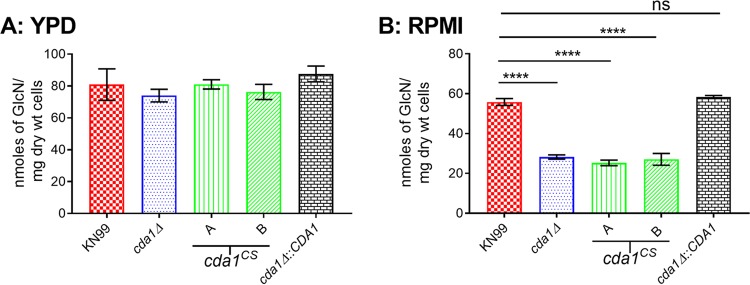
C. neoformans Cda1 catalytic point mutant strains fail to produce chitosan when grown under host-mimicking conditions. (A) Yeast strains were grown in YPD at 30°C for 72 h. Cells were collected, washed, and used for the measurement of cell wall chitosan by the MBTH assay. (B) The indicated strains were initially grown in YPD for 36 h, then collected, washed, and inoculated at a cell density of 500 cells/μl in RPMI medium containing 10% FBS, and incubated for 5 days at 37°C and 5% CO_2_. At the end of incubation, cells were collected, washed, and used for chitosan measurement by the MBTH assay. Data are the averages for three biological experiments with each experiment having two technical replicates. Significant differences between the groups were compared by one-way ANOVA, followed by Bonferroni’s multiple-comparison test (****, *P* < 0.0001 comparing chitosan levels of strain KN99 to each other strain).

The *cda1Δ* single deletion strain exhibits decreased levels of cell wall chitosan *in vitro* when cultured under host-mimicking conditions (Fig. 2B). Therefore, we wanted to determine whether expression of the *cda1*^CS^ allele in a background with Cda2 and Cda3 had a similar effect on the cell wall chitosan under host-mimicking conditions. We measured chitosan levels of two independent isolates of *cda1*^CS^ (isolates A and B) after growing the cells in either YPD or RPMI containing 10% FBS at 5% CO_2_ and 37°C. The KN99, *cda1Δ*, and *cda1Δ*::*CDA*1 strains served as control strains. We observed no significant differences in the amount of cell wall chitosan between strains when grown in YPD medium (Fig. 7A). However, we found significant decreases in chitosan for both isolates (isolates A and B) of the *cda1*^CS^ strain and for the *cda1Δ* strain compared to controls when grown in RPMI containing 10% FBS at 5% CO_2_ and 37°C (Fig. 7B). These data validate the hypothesis that in C. neoformans, Cda1 has a major role in the biosynthesis of chitosan under conditions that partially simulate host conditions, and the mutation of the conserved catalytic site residues eliminates its chitin deacetylase enzyme activity.

**FIG 8:**
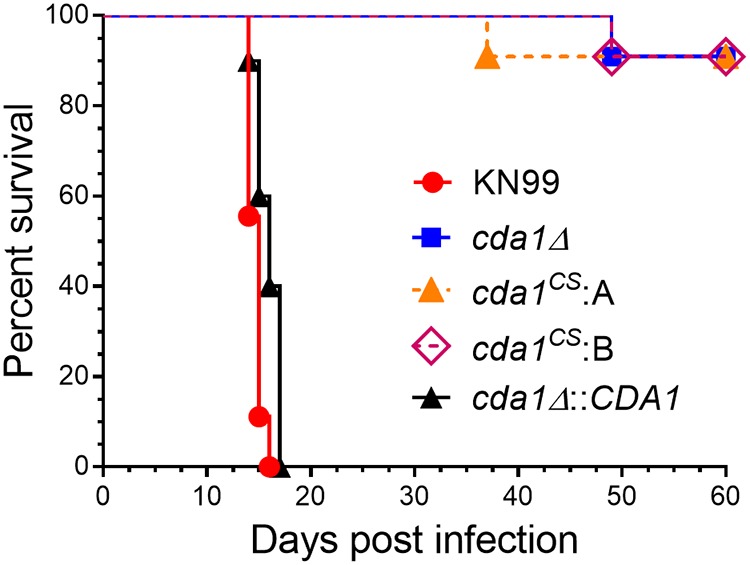
Chitin deacetylase activity of C. neoformans Cda1 is critical for fungal pathogenesis. Mice (female CBA/J; 4 to 6 weeks old) were inoculated with 10^5^ CFU of the indicated yeast strains by intranasal instillation. Mice were monitored for up to 60 days PI for survival by monitoring their body weight. Animals that lost 20% of their weight during inoculation were considered moribund and were euthanized. Data for each strain (*n* = 10 mice) are the combined results of two experiments, each with five animals. Survival curves were compared by log rank (Mantel-Cox) test (*P < *0.0001 comparing strain KN99 to *cda1Δ* or *cda1^CS^* isolate A or *cda1^CS^* isolate B).

We next tested the virulence of the catalytic point mutant strains in a mouse inhalation model. The point mutations of the catalytic site of Cda1 protein rendered the mutant strain severely attenuated for virulence, similar to the *cda1Δ* strain (Fig. 8). We measured the fungal burden in the lungs of the surviving mice at the endpoint of the survival experiment (60 days PI) and found similar fungal burdens in the lungs of the mice infected with *cda1*^CS^ strains compared to those infected with a *cda1Δ* strain (data not shown). These results strongly suggest that the mere presence of Cda1 protein is not enough to cause fungal pathogenesis but that its chitin deacetylase enzyme activity is essential for virulence in this model.

### C. neoformans *CDA1* is specifically upregulated in cells growing in the mouse lung.

**FIG 9:**
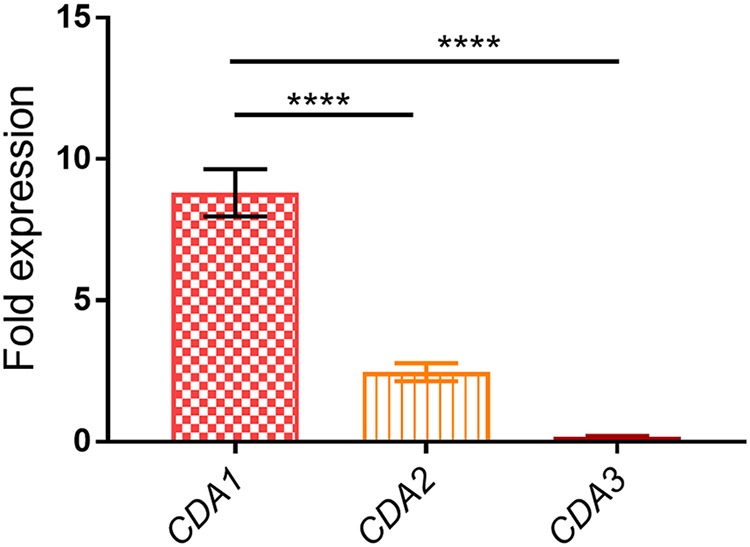
C. neoformans *CDA1* is transcriptionally upregulated during host infection. Mice were inoculated with 10^7^ CFU of KN99 cells. At day 7 PI, the lungs were excised and homogenized, and fungal cells were harvested and used for the isolation of total RNA. Total RNA (0.5 µg) was used for the synthesis of cDNA, which was subsequently subjected to quantitative real-time PCR using *CDA1*-specific primers. C. neoformans 18S rRNA transcript levels were used as a reference gene. Transcript levels of the respective genes in the cells used as inoculum served as control. Data are the averages for two independent experiments each with three animals per group. Fold expression was calculated for each gene comparing the extent of its upregulation in the lung samples to YPD-grown samples. Significant differences in the expression levels between genes were compared by ordinary one-way ANOVA, followed by Dunnett’s multiple-comparison test. (****, *P < *0.0001 comparing fold expression of *CDA1*, *CDA2*, and *CDA3* in the lung samples to their respective levels in the inoculum sample grown in YPD).

In order to begin to understand the mechanism of Cda1-mediated chitosan production during infection, we measured the transcriptional response of Cdas during a pulmonary infection. We inoculated mice with 10^7^ CFU of strain KN99 and harvested the fungal cells from the lung on day 7 PI. We monitored the Cda expression levels using quantitative PCR and compared RNA levels of the individual Cda genes to their levels in the inoculum, which was grown in YPD for 2 days. The *CDA1* transcript was highly upregulated (∼9-fold), CDA2 was modestly increased (2.5-fold), and CDA3 showed no change relative to their levels in the YPD inoculum (Fig. 9). These results support the hypothesis that growth in the lung environment of the infected animal stimulates specific upregulation of *CDA1*, which in turn correlates with Cda1 being the major contributor to chitosan biosynthesis.

## DISCUSSION

C. neoformans grows vegetatively as a budding yeast and has a defined sexual cycle that culminates in the production of basidiospores. An infection with C. neoformans can be initiated with either vegetative yeast or basidiospores (40). The yeast cell wall of C. neoformans contains considerable amounts of chitosan (11, 13). While chitosan is found in the walls of the ascospores in the nonpathogenic yeast S. cerevisiae, it is unknown whether chitosan is present in the basidiospore wall of C. neoformans (29–31). However, inhaled spores germinate and differentiate into the yeast form, and it is in this form that either infection will proceed or the yeast will be cleared from the host. Cell wall chitosan is dispensable when cryptococcal cells are cultured in standard growth medium, but chitosan is necessary for growth during infection (11–13, 41). Therefore, our goal was to identify and characterize the role of chitin deacetylases during a pulmonary infection. We have shown that despite having three functional chitin deacetylases, C. neoformans increases Cda1 transcription and employs Cda1 specifically to augment the synthesis of cell wall chitosan in a mammalian host through its chitin deacetylase enzyme activity; the chitosan produced by Cda1 is important for *Cryptococcus* to proliferate in the host lung.

The inability of the catalytic site mutants generated in the *cda2Δ3Δ* background to produce chitosan while still expressing the mutant Cda1 protein clearly indicated that we specifically targeted the chitin deacetylase activity of Cda1. Moreover, two independent isolates of *cda2Δ3Δcda1^CS^* exhibited *in vitro* phenotypes, which were identical to those of the *cda1Δ2Δ3Δ* strain, thus confirming the complete lack of Cda1 enzyme activity in *cda1^CS^* mutant strains. The *cda1^CS^* strain also exhibited phenotypes similar to those of the *cda1Δ* strain both in their failure to produce chitosan when grown in RPMI/10% FBS and 5% CO_2_ conditions and their inability to cause severe infection in the lungs of CBA/J mice. This is the first report in which the biosynthesis of chitosan and maintaining its normal levels in the cell wall can be directly implicated with fungal virulence.

Chitin deacetylases from several fungi have been reported to be active at diverse locations (42–45). Very little is known about the activity of chitin deacetylases in C. neoformans. In a previous report using a C. neoformans Cda2-specific monoclonal antibody and a cell fractionation procedure, it was revealed that Cda2 is present in the cytosol, membrane, cell wall, and secreted fractions (36). Although Cda2 was found in all of the fractions, chitosan synthesis by Cda2 requires its membrane localization (36). Therefore, in this study of Cda1, we wanted to make sure that mutation of its catalytic site did not affect either the stability or localization of the mutant protein. Fortunately, we found that the catalytic mutant form of Cda1 (*cda1^CS^*) was stably expressed and enriched in the membrane fraction like its native Cda1counterpart. We did observe that the total cell lysates prepared from mutant strains generated in the *cda2Δ3Δ* background showed decreased intensity in the activated stain-free gels for total protein (Fig. 4D). This might be due to the presence of a significant amount of capsular material present in these lysates interfering with the protein separation and staining. We have observed similar patterns for other chitosan-deficient strains such as *cda1Δ2Δ3Δ*, *chs3Δ*, and *csr*2*Δ* strains (unpublished results). In addition, the reported bigger capsules produced by the chitosan-deficient strains under vegetative growth conditions may have contributed to the poor resolution/staining of the proteins produced from mutant strains generated in the *cda2Δ3Δ* background (Fig. 4D) (12). Different metal ions have been reported to activate chitin deacetylase enzyme activity. The type of the metal ion required for enzyme activation is dependent on the enzyme source. We found that mutation of the putative metal binding site interfered with the protein stability of Cda1 in both the wild-type and *cda2Δ3Δ* background. This is consistent with the reports that metal ions of metalloproteins are important for the proper folding and stability of the final folded proteins (46). The chitin deacetylase genes are each expressed during vegetative growth, and each has a redundant role in the production of chitosan (12). However, it is striking to see that Cda1 plays a major role during growth in the infected lung.

It is interesting to note that at the end of the survival experiment, we did observe fungal burden in the lung even though the animals looked very healthy, were active, and gaining normal weight. The deletion of Cda1 did not affect the dissemination of the yeast cells in the infected animal, since we did observe fungal burden in the brains of some of the animals at the endpoint of survival experiments. This suggests that the host immune system was able to arrest the rapid proliferation of the mutant cells but failed to completely clear them. That there was substantial fungal burden in the brains of some of the animals infected with the *cda1Δ* strain at the endpoint of the survival experiment suggests that *cda1Δ* cells were able to exit the lung and either proliferate in the blood or cross the blood brain barrier (BBB) and proliferate in the brain. We did not see any correlation between the lung fungal burden and the corresponding appearance of yeast cells in the brain, suggesting that the presence of fungal cells in the brain did not require high CFU in the lung. In one of the animals, even though the lung CFU was decreased by fivefold compared to the inoculum level, it still harbored significant CFU in the brain.

Previous studies demonstrated that recombinant Cda1 when used with glucan particles induced an effective protective immunity to *Cryptococcus* challenge infection (34). We reasoned that the presence of Cda1 protein in the *cda1^CS^* mutants might negatively influence the intensity of virulence compared to the virulence of the *cda1Δ* strain. However, we did not see any difference in either the virulence or lung fungal burden at the endpoint of the survival experiment for *cda1Δ* and *cda1^CS^* infection. The amount of C*da1^CS^* protein present in *cda1^CS^* strain may not be sufficient enough to induce anticryptococcal host responses.

We have seen previously that chitosan-deficient cells are rapidly cleared from the host under certain conditions (13, 41). The loss of Cda1 does not render the cells completely devoid of chitosan either when they are growing in the infected lung or when they were incubated in culture under conditions that mimic the host. This suggests that the presence of chitosan in the cell wall protects yeast cells from host clearance. The level of chitosan in the cell wall is dynamic even when the cells are growing in YPD conditions either for wild-type cells or for *cda1*Δ cells. Therefore, it is possible that due to the absence of Cda1, *cda1*Δ cells may have either less chitosan or have a chitosan with a diminished degree of deacetylation that may render them sensitive to host chitinases. This uneven clearance of fungal cells in the lung may have contributed to the observed persistence of fungal burden.

We have shown that loss of chitosan transforms C. neoformans cells into a strong stimulator of host immune responses. The lungs of mice infected with 10^7^ CFU of the *cda1Δ2Δ3Δ* strain displayed a robust inflammatory response followed by complete clearance of fungal cells (41). This suggests that the ability of the fungal cells to convert chitin to chitosan is critical for masking the cell wall chitin from being recognized by host chitinases. This is not unique to C. neoformans, since other plant-pathogenic fungi have been shown to employ chitin-to-chitosan conversion as one of the mechanisms to prevent host immune activation during infection. Recently, it has been shown that Cda activity induces appressorium differentiation in the rice blast fungus Magnaporthe oryzae. It is surprising that despite *M. oryzae* encoding seven putative chitin deacetylases, only one Cda (Cbp1) was critical for converting chitin to chitosan during appressorium differentiation (47). In the tomato leaf mold Cladosporium fulvum, novel chitin binding proteins have been discovered to bind chitin during plant infection to mask chitin, thereby limiting triggering of plant immune responses (48, 49). These examples are plant pathogens, but given the presence of chitinases in mammalian hosts, this strategy could be effective for animal pathogens as well.

In C. neoformans, Cda2 and Cda3 appear to compensate for the loss of Cda1 when strains are cultured in YPD medium but fail to do so during infection of CBA/J mice. Our results suggest that production of chitosan during a pulmonary infection is largely dependent on the level of Cda1 transcription. Transcriptional regulation of Cda expression and Cda activity was initially found for S. cerevisiae, where the transcription of its *CDA1* and *CDA2* genes is restricted to sporulation (29, 50). C. neoformans transcription factor RIM101, a highly conserved pH response regulator, has been shown to negatively affect cell wall remodeling in the yeast growing either in the infected lung or under tissue culture conditions (Dulbecco’s modiﬁed Eagle’s medium [DMEM] with 5% CO_2_ at 37°C). RIM101 was found to regulate the expression of eight of the twelve genes that are predicted to play a role in the synthesis of chitin and chitosan. Under tissue culture conditions, Rim101 was found to bind the CDA1 promoter suggesting that Cda1 expression is regulated by Rim101. However, unlike *cda1Δ*, deletion of RIM101 did not attenuate C. neoformans virulence, suggesting that factors other than RIM101 may play an important role in the regulation of Cda1-mediated chitosan production (51–53). Further characterization of the *CDA1* promoter may provide insights into the roles of transcription factors and signaling pathways that regulate chitosan biosynthesis. In conclusion, understanding the mechanisms of chitosan biosynthesis and its regulation during infection is important and is a novel avenue for developing therapeutic molecules against cryptococcal infections.

## MATERIALS AND METHODS

### Fungal strains and media.

KN99α, a strain of C. neoformans serotype A (54), was used as the wild-type strain and as progenitor of mutant strains. Strains are listed in Table 1. Strains were grown on YPD (1% yeast extract, 2% bacto-peptone, and 2% dextrose). Solid media contained 2% bacto-agar. Selective YPD medium contained 100 μg/ml nourseothricin (NAT) (Werner BioAgents, Germany) and/or 200 μg/ml G418 (Geneticin; Gibco Life Technologies, USA). RPMI 1640 (catalog no. 10-040 CM; Corning) contained 10% fetal bovine serum (FBS) (catalog no. 26140; Gibco-Thermo Fisher Technologies).

**TABLE 1:** Fungal strains used in this study

Strain	Resistance marker	Strain background	Reference or source
KN99			54
*cda1Δ*	HYG	KN99	12
*cda2Δ*	NAT	KN99	12
*cda3Δ*	G418	KN99	12
*cda2Δ3Δ*	NAT/Phleo		12
*cda1Δ*::*CDA1*	G418	*cda1Δ*	This study (RUCN1219)
*cda1^D166N^*^,^*^R254A^*^,^*^D294N^* (*cda1*^CS^)	G418	*cda1Δ*	This study (RUCN1218)
*cda1^D167N^*^,^*^H216A^*^,^*^H220A^* (*cda1*^MB^)	G418	*cda1Δ*	This study (RUCN1220)
*cda2Δ3Δcda1^D166N^*^,^*^R254A^*^,^*^D294N^*	NAT/Phleo/G418	*cda2Δ3Δ*	This study (JLCN 933)
*cda2Δ3Δcda1^D167N^*^,^*^H216A^*^,^*^H220A^*	NAT/Phleo/G418	*cda2Δ3Δ*	This study (JLCN 936)
*cda2Δ3Δ*::*CDA1*	NAT/Phleo/G418	*cda2Δ3Δ*	This study (JLCN 939)
*cda1Δcda2Δ3Δ*	HYG/NAT/Phleo	KN99	12

### Generation of predicted catalytic and metal binding mutant strains of Cda1.

A diagram that depicts the steps for making gene replacement constructs is shown in Fig. S1 in the supplemental material. Scheme A describes the steps taken to construct wild-type and mutant *CDA1* genes. Briefly, two separate 490-bp genomic DNA fragments (Cda fragment B) of C. neoformans *CDA1* (CNAG_05799) in which predicted catalytic and metal binding sites are located (encoding amino acids 154 to 302) were chemically synthesized by Integrated DNA Technologies, USA. Nucleic acid substitutions to generate mutations were introduced during gene synthesis. DNA regions that flanked Cda fragment B were amplified by PCR using gene-specific primers and KN99 genomic DNA as the template. The 5′ flanking PCR product, Cda gene fragment A, was 1,479 bp and included 750 bp of the Cda1 promoter and DNA encoding amino acids 1 to 153. The 3′ flanking PCR product, Cda gene fragment C, was 916 bp and included DNA encoding amino acids 303 to 470 and 306 bp of the 3′ untranslated region (UTR). All three *CDA1* gene fragments were assembled together using overlap PCR to generate wild-type or mutant versions of the full-length *CDA1* gene. The final PCR products of 2,885 bp were cloned as described below and sequenced to verify that no errors had been introduced during fusion and amplification.

### Construction of a plasmid to clone wild-type and mutant *CDA1* gene fragments.

Next, the *CDA1* DNA generated by PCR fusion described above was cloned into a vector that enabled gene replacement and selection of transformants on medium containing G418. First, a 809-bp genomic region encompassing a region 2,032 bp upstream of the Cda1 translation start site (ATG) was amplified from genomic DNA of strain KN99 to provide a 5′ homologous region for targeted integration. This 809-bp fragment was then fused to 2.9 kb of DNA encoding G418 resistance that had been amplified from the plasmid pMH12T employing In-Fusion HD cloning (TaKaRa Bio, USA) to generate the CDA15’homology+G418 fragment (55). Unique restriction sites were engineered in the primers for each fragment as shown in scheme B of Fig. S1. The CDA15’homology+G418 fragment was cloned into pMH12T at the NotI site after digesting pMH12T with HindIII and NotI. The NotI site was preserved, while cloning eliminated the HindIII restriction site to generate plasmid pCDA15prime+G418. Different versions of the Cda1 gene fragment (either wild type or mutant) were cloned at the unique PacI and NotI sites in the plasmid pCDA15prime+G418 to generate plasmids pCDA1A, pCDA1B, and pCDA1C containing wild-type, catalytic site mutant, and metal binding site mutant forms of Cda1, respectively. Finally, XhoI digestion of each of the plasmids was carried out to release the CDA1 gene fragment (containing sequences of the wild-type and the corresponding point mutant versions) as shown in scheme B for biolistic transformation. The strain created after transformation of a construct containing a wild-type gene copy was designated a *cda1Δ*::*CDA1* strain.

### Transformation and characterization of C. neoformans mutants.

Recipient strains of C. neoformans were transformed biolistically following the protocol described earlier (55, 56). Drug-resistant transformants that formed colonies in 3 to 5 days were passaged four times in liquid YPD medium before reselection of drug resistance on agar. Transformants were further characterized by diagnostic PCR of their genomic DNA using primers at the 5′ and 3′ junction of the integration site of the transforming DNA. Southern blot hybridizations were done to verify the absence of random DNA integrations as described previously (57).

### Evaluation of C. neoformans to stress under *in vitro* conditions.

Solid YPD medium was made with the desired amount of either SDS or NaCl. To subject cells to oxidative and nitrosative stress, YNB medium buffered to pH 4 with 1 M succinic acid was used with a final concentration of 1 mM H_2_O_2_ or 0.75 mM NaNO_2_, respectively. For plating, wild-type and mutant strains were grown in liquid YPD for 24 h at 30°C. Cells were diluted to an OD_650_ of 1.0, and 10-fold serial dilutions were made. Four microliters of each dilution were spotted on the plate, and the plates were incubated for 2 or 3 days at the appropriate temperature and photographed. Eosin Y staining was carried out as described earlier (12).

### Cellular chitosan measurement.

We optimized an MBTH (3-methyl-2-benzothiazolinone hydrazone) (catalog no. 65875; Sigma-Aldrich, USA)-based chemical method for the measurement of cell wall chitosan by the modification of a previously reported assay for the quantification of glycosaminoglycan hexosamine (58). C. neoformans cells were collected by centrifugation and washed with distilled water, and the cell pellets were lyophilized to measure their dry weight. The lyophilized material was suspended in 10 ml of 6% (wt/vol) KOH (potassium hydroxide) and incubated at 80°C in a water bath for 60 min with occasional mixing of the cell suspension. The KOH-treated material was washed three times with PBS, pH 7.4, and adjusted to 10 mg (dry weight)/ml PBS. For cells grown in RPMI medium, the KOH-treated material was sonicated into suspension using a probe sonicator (Fisher sonic dismembrator model 300 attached to GraLab model 451 high-accuracy digital electronic timer): 5 cycles of 60 s. A 0.1-ml aliquot of the alkali-treated material was used for the chitosan measurement as described next.

The 0.1-ml sample was mixed with 0.1 ml of 1 M HCl, followed by the addition of 400 µl of freshly prepared 2.5% (wt/vol) sodium nitrite (catalog no. 237213; Sigma-Aldrich) with vortexing to mix. The deamination and depolymerization reactions were continued for 15 min at 25°C. Next, 200 µl of 12.5% (wt/vol) ammonium sulfamate (catalog no. 228745; Sigma-Aldrich) was added slowly to neutralize unreacted sodium nitrite. After 5 min of incubation at 25°C, 200 µl of 0.25% (wt/vol) MBTH prepared in water was added, and the reaction mixture was incubated at 37°C for 30 min. After the incubation, 200 µl of 0.5% ferric chloride (catalog no. 44944; Sigma-Aldrich) in water (wt/vol) was added, and the contents were mixed and then incubated at 37°C for 5 min. Finally, the reaction mixture was centrifuged at 12,000 × *g* for 5 min, and the absorbance of the supernatant was measured at 650 nm. Standard curves were prepared from stocks of D-glucosamine hydrochloride (catalog no. G4875; Sigma-Aldrich) in the concentration range of 10 to 100 nmol. The amount of cellular chitosan was expressed as nanomoles of glucosamine monomer per milligram (dry weight) of the sample.

### Isolation of C. neoformans cells from infected murine lungs for chitosan measurements.

Animals were infected intranasally with 10^7^ colony-forming units (CFU) of desired strains after growing them in YPD (50 ml) for 2 days at 300 rpm and 30°C. On day 7 postinfection (PI), the lungs of the infected animals were excised and homogenized (PRO 200 homogenizer; Midsci, USA) in 2 ml of PBS. Homogenized lung tissue was extracted by the addition of 8 ml of 6% (wt/vol) KOH and incubation at 80°C for 60 min. After the incubation, tissue homogenate was centrifuged at 3,750 × *g* for 10 min at 25°C. The pellet was suspended in 2 ml of PBS and passed sequentially through 100-µm and then 40-µm nylon filters to remove insoluble tissue materials. After two rounds of washing with PBS (pH 7.4), pellets were suspended in 400 µl of PBS. The suspension was disrupted further by probe sonication as described above, and the number of *Cryptococcus* cells was counted using a hemocytometer and an Olympus CKX41 inverted microscope. Samples were subjected to chitosan measurement by the MBTH assay as described above. The amount of chitosan was expressed as nanomoles of glucosamine per 10^7^ yeast cells.

### Virulence and fungal burden assays.

C. neoformans strains were grown at 30°C and 300 rpm for 2 days in 50 ml YPD. The cells were centrifuged, washed in endotoxin-free 1× PBS, and suspended in 5 ml of the same. The cells were counted with a hemocytometer and diluted to 2 × 10^6^ cells ml^−1^. CBA/J female mice (Jackson Laboratories) were anesthetized with an intraperitoneal injection (200 µl) of ketamine (8 mg/ml)/dexdomitor (0.05 mg/ml) mixture which was reversed by an intraperitoneal injection of antisedan (200 µl) (0.25 mg/ml). Mice were allowed to inhale 1 × 10^5^ cells in 50 μl, which were dripped into the nares (59). For virulence assays, the mice were weighed before and during the course of infection. Mice were sacrificed by CO_2_ asphyxiation if they reached 80% of their original body weight. At this point, the mice appeared morbidly ill, displaying a ruffled coat, lethargy, a hunched posture, unstable gait, and loss of appetite. For the determination of CFU, lung or brain from each mouse was placed in 2.0 ml of 1× PBS (pH 7), homogenized, serially diluted, plated onto YPD agar supplemented with 100 mg/ml streptomycin and 100 mg/ml ampicillin, and incubated for 2 days at 30°C. The total number of CFU per organ was calculated.

### Generation of C. neoformans Cda1-specific antibody and immunoblot analysis.

A recombinant protein of C. neoformans Cda1 (amino acids 20 to 374) was expressed in E. coli and purified as described previously (34). This protein was used as an antigen to generate affinity-purified polyclonal antibody through Thermo Fisher Scientific, IL, USA. The specificity of the antibody to Cda1 protein was confirmed by hybridization to cell lysates of various single Cda gene deletion strains (*cda1Δ*, *cda2Δ*, and *cda3Δ* strains). For the Western blot analysis, yeast cells were grown in 20 ml of YPD for 48 h. Cells were collected by centrifugation and washed two times with PBS at 4°C, and cell pellets were flash frozen and stored at −80°C. For the preparation of whole-cell lysate, each frozen cell pellet was suspended in 1.5 ml of lysis buffer (100 mM Tris-HCl [pH 8] containing 1% sodium deoxycholate, 1% Triton X-100, 0.1% SDS, 1 mM phenylmethylsulfonyl fluoride [PMSF], and cOmplete ULTRA tablet Mini EDTA-free [Roche Diagnostics, Germany]) in a 2-ml tube. Then, 0.5 ml of 0.5-mm zirconia beads (BioSpec Products, USA) was added, and the cells were broken using a minibead beater (Biospec Products, Bartlesville, OK) for eight cycles of 30 s with 2 min cooling on ice. Broken cells were centrifuged at 10,000 × *g* at 4°C, and the supernatant was collected and used as whole-cell lysate after mixing with sterile glycerol to a final concentration of 5% (vol/vol). Protein concentration was determined using a BCA protein assay kit (Thermo-Fisher, USA). Samples of 50 µg of protein were separated on a 12% Stain-Free SDS-polyacrylamide gel (Bio-Rad). Protein was transferred to a nitrocellulose membrane using a Bio-Rad protein transfer system. Blots were blocked in 5% milk containing 0.2% BSA in Tris-buffered saline (pH 7.4) with 0.1% Tween 20. Cda1-specific antibody was used at a 1:4,000 dilution, and the blot was incubated overnight at 4°C. HRP-conjugated anti-rabbit IgG secondary antibody (catalog no. A6154; Sigma-Aldrich, USA) was used at a 1:5,000 dilution, and the blot was incubated for 1 h at 25°C. Finally, the blot was incubated with Clarity Western ECL substrate (catalog no. 170-5060; Bio-Rad, Hercules, CA), and the image was acquired through a ChemiDoc Touch Imaging system (Bio-Rad, Hercules, CA). Subcellular fractionation to determine the localization of Cda1 was carried out essentially as described by Gilbert et al. (36).

### Isolation of fungal cells from infected lung for RNA extraction.

CBA/J mice were infected intranasally as described above with 10^7^ CFU of strain KN99. At day 7 PI, lungs were excised and homogenized with 2 ml of water in the presence of 100 U of RNase inhibitor (catalog no. N8080119; ThermoFisher Scientific, USA) at 4°C. The lung homogenate was centrifuged, the pellet was washed two times with cold water with vortexing before being finally filtered through 100-µm and 40-µm nylon filters. As a control sample, KN99 cells from the culture used for infection was subjected to wash and filtration procedures used for lung homogenates. Isolated fungal cells were subjected to RNA purification as described previously using RNeasy plant minikit (Qiagen, GmbH, Germany) (57). Total RNA (0.5 µg) was used for first-strand cDNA synthesis using the iScript cDNA synthesis kit (Bio-Rad, Hercules, CA) per the manufacturer’s instructions. The resulting cDNA was used as a template in quantitative PCR using SsoFast EvaGreen Supermix (Bio-Rad, Hercules, CA) according to the manufacturer’s recommendations. The CFX96 real-time system (Bio-Rad, Hercules, CA) was used as the fluorescence detector with the following protocol for the PCR. The initial denaturation step was 30 s at 95°C, followed by 40 cycles, with 1 cycle consisting of 95°C for 5 s and 20 s at 60°C. A melting curve was measured at the end of each reaction to confirm that a single product was amplified.[Supplementary-material figS2][Supplementary-material tabS1]

10.1128/mBio.02087-18.2 FIG S2 Stain-free image of the blot used for the Western blot analysis in Fig. 5. Proteins in each fraction (total cell lysate [L], cytosolic fraction [C], membrane fraction [M], or cell wall fraction [CW]) were quantified by BCA protein assay, and equal amounts of protein were separated on 12% stain-free Tris-glycine gels. After the separation, gels were photo-activated, and the image was acquired through ChemiDoc Touch Imaging system (Bio-Rad Laboratories, USA). Download FIG S2, DOCX file, 8.4 MB. Copyright © 2018 Upadhya et al. 2018 Upadhya et al. This content is distributed under the terms of the Creative Commons Attribution 4.0 International license.

10.1128/mBio.02087-18.3 TABLE S1 Primers used in this study. Download Table S1, DOCX file, 0.1 MB. Copyright © 2018 Upadhya et al. 2018 Upadhya et al. This content is distributed under the terms of the Creative Commons Attribution 4.0 International license.

## References

[1] Rajasingham R, Smith RM, Park BJ, Jarvis JN, Govender NP, Chiller TM, Denning DW, Loyse A, Boulware DR 2017 Global burden of disease of HIV-associated cryptococcal meningitis: an updated analysis. Lancet Infect Dis 17:873–881. doi:10.1016/S1473-3099(17)30243-8. 28483415 PMC5818156

[2] Litvintseva AP, Carbone I, Rossouw J, Thakur R, Govender NP, Mitchell TG 2011 Evidence that the human pathogenic fungus *Cryptococcus neoformans* var. *grubii* may have evolved in Africa. PLoS One 6:e19688. doi:10.1371/journal.pone.0019688. 21589919 PMC3092753

[3] Litvintseva AP, Thakur R, Reller LB, Mitchell TG 2005 Prevalence of clinical isolates of *Cryptococcus gattii* serotype C among patients with AIDS in Sub-Saharan Africa. J Infect Dis 192:888–892. doi:10.1086/432486. 16088839

[4] Hiremath SS, Chowdhary A, Kowshik T, Randhawa HS, Sun S, Xu J 2008 Long-distance dispersal and recombination in environmental populations of *Cryptococcus neoformans* var. *grubii* from India. Microbiology 154:1513–1524. doi:10.1099/mic.0.2007/015594-0. 18451060

[5] Idnurm A, Bahn YS, Nielsen K, Lin X, Fraser JA, Heitman J 2005 Deciphering the model pathogenic fungus *Cryptococcus neoformans*. Nat Rev Microbiol 3:753–764. doi:10.1038/nrmicro1245. 16132036

[6] Park BJ, Wannemuehler KA, Marston BJ, Govender N, Pappas PG, Chiller TM 2009 Estimation of the current global burden of cryptococcal meningitis among persons living with HIV/AIDS. AIDS 23:525–530. doi:10.1097/QAD.0b013e328322ffac. 19182676

[7] Kidd SE, Hagen F, Tscharke RL, Huynh M, Bartlett KH, Fyfe M, Macdougall L, Boekhout T, Kwon-Chung KJ, Meyer W 2004 A rare genotype of *Cryptococcus gattii* caused the cryptococcosis outbreak on Vancouver Island (British Columbia, Canada). Proc Natl Acad Sci U S A 101:17258–17263. doi:10.1073/pnas.0402981101. 15572442 PMC535360

[8] Springer DJ, Billmyre RB, Filler EE, Voelz K, Pursall R, Mieczkowski PA, Larsen RA, Dietrich FS, May RC, Filler SG, Heitman J 2014 *Cryptococcus gattii* VGIII isolates causing infections in HIV/AIDS patients in Southern California: identification of the local environmental source as arboreal. PLoS Pathog 10:e1004285. doi:10.1371/journal.ppat.1004285. 25144534 PMC4140843

[9] Chau TT, Mai NH, Phu NH, Nghia HD, Chuong LV, Sinh DX, Duong VA, Diep PT, Campbell JI, Baker S, Hien TT, Lalloo DG, Farrar JJ, Day JN 2010 A prospective descriptive study of cryptococcal meningitis in HIV uninfected patients in Vietnam - high prevalence of *Cryptococcus neoformans* var *grubii* in the absence of underlying disease. BMC Infect Dis 10:199. doi:10.1186/1471-2334-10-199. 20618932 PMC2910700

[10] Free SJ 2013 Fungal cell wall organization and biosynthesis. Adv Genet 81:33–82. doi:10.1016/B978-0-12-407677-8.00002-6. 23419716

[11] Banks IR, Specht CA, Donlin MJ, Gerik KJ, Levitz SM, Lodge JK 2005 A chitin synthase and its regulator protein are critical for chitosan production and growth of the fungal pathogen *Cryptococcus neoformans*. Eukaryot Cell 4:1902–1912. doi:10.1128/EC.4.11.1902-1912.2005. 16278457 PMC1287864

[12] Baker LG, Specht CA, Donlin MJ, Lodge JK 2007 Chitosan, the deacetylated form of chitin, is necessary for cell wall integrity in *Cryptococcus neoformans*. Eukaryot Cell 6:855–867. doi:10.1128/EC.00399-06. 17400891 PMC1899242

[13] Baker LG, Specht CA, Lodge JK 2011 Cell wall chitosan is necessary for virulence in the opportunistic pathogen *Cryptococcus neoformans*. Eukaryot Cell 10:1264–1268. doi:10.1128/EC.05138-11. 21784998 PMC3187048

[14] Lee CG, Da Silva CA, Dela Cruz CS, Ahangari F, Ma B, Kang MJ, He CH, Takyar S, Elias JA 2011 Role of chitin and chitinase/chitinase-like proteins in inflammation, tissue remodeling, and injury. Annu Rev Physiol 73:479–501. doi:10.1146/annurev-physiol-012110-142250. 21054166 PMC3864643

[15] Rohatgi S, Pirofski LA 2015 Host immunity to *Cryptococcus neoformans*. Future Microbiol 10:565–581. doi:10.2217/fmb.14.132. 25865194 PMC4523559

[16] Voelz K, May RC 2010 Cryptococcal interactions with the host immune system. Eukaryot Cell 9:835–846. doi:10.1128/EC.00039-10. 20382758 PMC2901644

[17] Brown SM, Campbell LT, Lodge JK 2007 *Cryptococcus neoformans*, a fungus under stress. Curr Opin Microbiol 10:320–325. doi:10.1016/j.mib.2007.05.014. 17707685 PMC2570326

[18] Upadhya R, Campbell LT, Donlin MJ, Aurora R, Lodge JK 2013 Global transcriptome profile of *Cryptococcus neoformans* during exposure to hydrogen peroxide induced oxidative stress. PLoS One 8:e55110. doi:10.1371/journal.pone.0055110. 23383070 PMC3557267

[19] Bahn YS, Jung KW 2013 Stress signaling pathways for the pathogenicity of Cryptococcus. Eukaryot Cell 12:1564–1577. doi:10.1128/EC.00218-13. 24078305 PMC3889573

[20] Fries BC, Goldman DL, Cherniak R, Ju R, Casadevall A 1999 Phenotypic switching in *Cryptococcus neoformans* results in changes in cellular morphology and glucuronoxylomannan structure. Infect Immun 67:6076–6083. 10531269 10.1128/iai.67.11.6076-6083.1999 PMC96995

[21] Rodrigues ML, Nakayasu ES, Oliveira DL, Nimrichter L, Nosanchuk JD, Almeida IC, Casadevall A 2008 Extracellular vesicles produced by *Cryptococcus neoformans* contain protein components associated with virulence. Eukaryot Cell 7:58–67. doi:10.1128/EC.00370-07. 18039940 PMC2224146

[22] Doering TL, Nosanchuk JD, Roberts WK, Casadevall A 2008 Melanin as a potential cryptococcal defence against microbicidal proteins. Med Mycol 37:175–181. doi:10.1111/j.1365-280X.1999.00218.x. 10421849

[23] Wang Y, Aisen P, Casadevall A 1995 *Cryptococcus neoformans* melanin and virulence: mechanism of action. Infect Immun 63:3131–3136. 7622240 10.1128/iai.63.8.3131-3136.1995 PMC173427

[24] Liu L, Tewari RP, Williamson PR 1999 Laccase protects *Cryptococcus neoformans* from antifungal activity of alveolar macrophages. Infect Immun 67:6034–6039. 10531264 10.1128/iai.67.11.6034-6039.1999 PMC96990

[25] Punja ZK, Zhang YY 1993 Plant chitinases and their roles in resistance to fungal diseases. J Nematol 25:526–540. 19279806 PMC2619419

[26] Springer DJ, Mohan R, Heitman J 2017 Plants promote mating and dispersal of the human pathogenic fungus Cryptococcus. PLoS One 12:e0171695. doi:10.1371/journal.pone.0171695. 28212396 PMC5315327

[27] Mayer FL, Kronstad JW 2017 Disarming fungal pathogens: *Bacillus safensis* inhibits virulence factor production and biofilm formation by *Cryptococcus neoformans* and *Candida albicans*. mBio 8:e01537-17. doi:10.1128/mBio.01537-17. 28974618 PMC5626971

[28] Steenbergen JN, Shuman HA, Casadevall A 2001 *Cryptococcus neoformans* interactions with amoebae suggest an explanation for its virulence and intracellular pathogenic strategy in macrophages. Proc Natl Acad Sci U S A 98:15245–15250. doi:10.1073/pnas.261418798. 11742090 PMC65014

[29] Christodoulidou A, Bouriotis V, Thireos G 1996 Two sporulation-specific chitin deacetylase-encoding genes are required for the ascospore wall rigidity of *Saccharomyces cerevisiae*. J Biol Chem 271:31420–31425. doi:10.1074/jbc.271.49.31420. 8940152

[30] Christodoulidou A, Briza P, Ellinger A, Bouriotis V 1999 Yeast ascospore wall assembly requires two chitin deacetylase isozymes. FEBS Lett 460:275–279. doi:10.1016/S0014-5793(99)01334-4. 10544249

[31] Coluccio A, Neiman AM 2004 Interspore bridges: a new feature of the *Saccharomyces cerevisiae* spore wall. Microbiology 150:3189–3196. doi:10.1099/mic.0.27253-0. 15470099

[32] Ohtakara A, Izume M, Mitsutomi M 1988 Action of microbial chitinases on chitosan with different degrees of deacetylation. Agric Biol Chem 52:3181–3182. doi:10.1080/00021369.1988.10869204.

[33] Levitz SM, Nong S, Mansour MK, Huang C, Specht CA 2001 Molecular characterization of a mannoprotein with homology to chitin deacetylases that stimulates T cell responses to *Cryptococcus neoformans*. Proc Natl Acad Sci U S A 98:10422–10427. doi:10.1073/pnas.181331398. 11504924 PMC56976

[34] Specht CA, Lee CK, Huang H, Hester MM, Liu J, Luckie BA, Torres Santana MA, Mirza Z, Khoshkenar P, Abraham A, Shen ZT, Lodge JK, Akalin A, Homan J, Ostroff GR, Levitz SM 2017 Vaccination with recombinant Cryptococcus proteins in glucan particles protects mice against cryptococcosis in a manner dependent upon mouse strain and cryptococcal species. mBio 8:e01872-17. doi:10.1128/mBio.01872-17. 29184017 PMC5705919

[35] Biondo C, Beninati C, Bombaci M, Messina L, Mancuso G, Midiri A, Galbo R, Teti G 2003 Induction of T helper type 1 responses by a polysaccharide deacetylase from *Cryptococcus neoformans*. Infect Immun 71:5412–5417. doi:10.1128/IAI.71.9.5412-5417.2003. 12933895 PMC187367

[36] Gilbert NM, Baker LG, Specht CA, Lodge JK 2012 A glycosylphosphatidylinositol anchor is required for membrane localization but dispensable for cell wall association of chitin deacetylase 2 in *Cryptococcus neoformans*. mBio 3:e00007-12. doi:10.1128/mBio.00007-12. 22354955 PMC3280450

[37] Blair DE, Schuttelkopf AW, MacRae JI, van Aalten DM 2005 Structure and metal-dependent mechanism of peptidoglycan deacetylase, a streptococcal virulence factor. Proc Natl Acad Sci U S A 102:15429–15434. doi:10.1073/pnas.0504339102. 16221761 PMC1252587

[38] Blair DE, Hekmat O, Schuttelkopf AW, Shrestha B, Tokuyasu K, Withers SG, van Aalten DM 2006 Structure and mechanism of chitin deacetylase from the fungal pathogen *Colletotrichum lindemuthianum*. Biochemistry 45:9416–9426. doi:10.1021/bi0606694. 16878976

[39] Rivero-Gutiérrez B, Anzola A, Martínez-Augustin O, de Medina FS 2014 Stain-free detection as loading control alternative to Ponceau and housekeeping protein immunodetection in Western blotting. Anal Biochem 467:1–3. doi:10.1016/j.ab.2014.08.027. 25193447

[40] Giles SS, Dagenais TR, Botts MR, Keller NP, Hull CM 2009 Elucidating the pathogenesis of spores from the human fungal pathogen *Cryptococcus neoformans*. Infect Immun 77:3491–3500. doi:10.1128/IAI.00334-09. 19451235 PMC2715683

[41] Upadhya R, Lam WC, Maybruck B, Specht CA, Levitz SM, Lodge JK 2016 Induction of protective immunity to cryptococcal infection in mice by a heat-killed, chitosan-deficient strain of *Cryptococcus neoformans*. mBio 7:e00547-16. doi:10.1128/mBio.00547-16. 27165801 PMC4959652

[42] Gao XD, Katsumoto T, Onodera K 1995 Purification and characterization of chitin deacetylase from *Absidia coerulea*. J Biochem 117:257–263. doi:10.1093/jb/117.2.257. 7608108

[43] Martinou A, Koutsioulis D, Bouriotis V 2002 Expression, purification, and characterization of a cobalt-activated chitin deacetylase (Cda2p) from *Saccharomyces cerevisiae*. Protein Expr Purif 24:111–116. doi:10.1006/prep.2001.1547. 11812231

[44] Alfonso C, Nuero OM, Santamaria F, Reyes F 1995 Purification of a heat-stable chitin deacetylase from *Aspergillus nidulans* and its role in cell wall degradation. Curr Microbiol 30:49–54. doi:10.1007/BF00294524. 7765883

[45] Chibucos MC, Soliman S, Gebremariam T, Lee H, Daugherty S, Orvis J, Shetty AC, Crabtree J, Hazen TH, Etienne KA, Kumari P, O’Connor TD, Rasko DA, Filler SG, Fraser CM, Lockhart SR, Skory CD, Ibrahim AS, Bruno VM 2016 An integrated genomic and transcriptomic survey of mucormycosis-causing fungi. Nat Commun 7:12218. doi:10.1038/ncomms12218. 27447865 PMC4961843

[46] Tainer JA, Roberts VA, Getzoff ED 1992 Protein metal-binding sites. Curr Opin Biotechnol 3:378–387. doi:10.1016/0958-1669(92)90166-G. 1368439

[47] Kuroki M, Okauchi K, Yoshida S, Ohno Y, Murata S, Nakajima Y, Nozaka A, Tanaka N, Nakajima M, Taguchi H, Saitoh KI, Teraoka T, Narukawa M, Kamakura T 2017 Chitin-deacetylase activity induces appressorium differentiation in the rice blast fungus *Magnaporthe oryzae*. Sci Rep 7:9697. doi:10.1038/s41598-017-10322-0. 28852173 PMC5575296

[48] Bolton MD, van Esse HP, Vossen JH, de Jonge R, Stergiopoulos I, Stulemeijer IJ, van den Berg GC, Borras-Hidalgo O, Dekker HL, de Koster CG, de Wit PJ, Joosten MH, Thomma BP 2008 The novel *Cladosporium fulvum* lysin motif effector Ecp6 is a virulence factor with orthologues in other fungal species. Mol Microbiol 69:119–136. doi:10.1111/j.1365-2958.2008.06270.x. 18452583

[49] de Jonge R, van Esse HP, Kombrink A, Shinya T, Desaki Y, Bours R, van der Krol S, Shibuya N, Joosten MH, Thomma BP 2010 Conserved fungal LysM effector Ecp6 prevents chitin-triggered immunity in plants. Science 329:953–955. doi:10.1126/science.1190859. 20724636

[50] Mishra C, Semino CE, McCreath KJ, de la Vega H, Jones BJ, Specht CA, Robbins PW 1997 Cloning and expression of two chitin deacetylase genes of *Saccharomyces cerevisiae*. Yeast 13:327–336. doi:10.1002/(SICI)1097-0061(19970330)13:4<327::AID-YEA96>3.0.CO;2-T. 9133736

[51] O’Meara TR, Holmer SM, Selvig K, Dietrich F, Alspaugh JA 2013 *Cryptococcus neoformans* Rim101 is associated with cell wall remodeling and evasion of the host immune responses. mBio 4:e00522-12. doi:10.1128/mBio.00522-12. 23322637 PMC3551547

[52] O’Meara TR, Norton D, Price MS, Hay C, Clements MF, Nichols CB, Alspaugh JA 2010 Interaction of *Cryptococcus neoformans* Rim101 and protein kinase A regulates capsule. PLoS Pathog 6:e1000776. doi:10.1371/journal.ppat.1000776. 20174553 PMC2824755

[53] O’Meara TR, Xu W, Selvig KM, O’Meara MJ, Mitchell AP, Alspaugh JA 2014 The *Cryptococcus neoformans* Rim101 transcription factor directly regulates genes required for adaptation to the host. Mol Cell Biol 34:673–684. doi:10.1128/MCB.01359-13. 24324006 PMC3911494

[54] Nielsen K, Cox GM, Wang P, Toffaletti DL, Perfect JR, Heitman J 2003 Sexual cycle of *Cryptococcus neoformans* var. *grubii* and virulence of congenic a and alpha isolates. Infect Immun 71:4831–4841. doi:10.1128/IAI.71.9.4831-4841.2003. 12933823 PMC187335

[55] Hua J, Meyer JD, Lodge JK 2000 Development of positive selectable markers for the fungal pathogen *Cryptococcus neoformans*. Clin Diagn Lab Immunol 7:125–128. doi:10.1128/CDLI.7.1.125-128.2000. 10618292 PMC95837

[56] Toffaletti DL, Rude TH, Johnston SA, Durack DT, Perfect JR 1993 Gene transfer in *Cryptococcus neoformans* by use of biolistic delivery of DNA. J Bacteriol 175:1405–1411. doi:10.1128/jb.175.5.1405-1411.1993. 8444802 PMC193227

[57] Upadhya R, Lam WC, Maybruck BT, Donlin MJ, Chang AL, Kayode S, Ormerod KL, Fraser JA, Doering TL, Lodge JK 2017 A fluorogenic *C. neoformans* reporter strain with a robust expression of m-cherry expressed from a safe haven site in the genome. Fungal Genet Biol 108:13–25. doi:10.1016/j.fgb.2017.08.008. 28870457 PMC5681388

[58] Smith RL, Gilkerson E 1979 Quantitation of glycosaminoglycan hexosamine using 3-methyl-2-benzothiazolone hydrazone hydrochloride. Anal Biochem 98:478–480. doi:10.1016/0003-2697(79)90170-2. 496014

[59] Cox GM, Mukherjee J, Cole GT, Casadevall A, Perfect JR 2000 Urease as a virulence factor in experimental cryptococcosis. Infect Immun 68:443–448. doi:10.1128/IAI.68.2.443-448.2000. 10639402 PMC97161

[60] Notredame C, Higgins DG, Heringa J 2000 T-Coffee: a novel method for fast and accurate multiple sequence alignment. J Mol Biol 302:205–217. doi:10.1006/jmbi.2000.4042. 10964570

[61] Di Tommaso P, Moretti S, Xenarios I, Orobitg M, Montanyola A, Chang JM, Taly JF, Notredame C 2011 T-Coffee: a web server for the multiple sequence alignment of protein and RNA sequences using structural information and homology extension. Nucleic Acids Res 39:W13–W17. doi:10.1093/nar/gkr245. 21558174 PMC3125728

