# Supplementary material for: Cryptococcus neoformans Cda1 and Its Chitin Deacetylase Activity Are Required for Fungal Pathogenesis
Source: mBio. 2018 Nov 20;9(6):e02087-18. doi: 10.1128/mBio.02087-18 (PMC6247093; doi:10.1128/mBio.02087-18)
Supplement: FIG S1 [file mbo006184181sf1.docx]

**Fig. S1**

**A**

Fragment A

Fragment B

Fragment C

*****

*****

*****

1

2

5

4

3

6

**B**

C


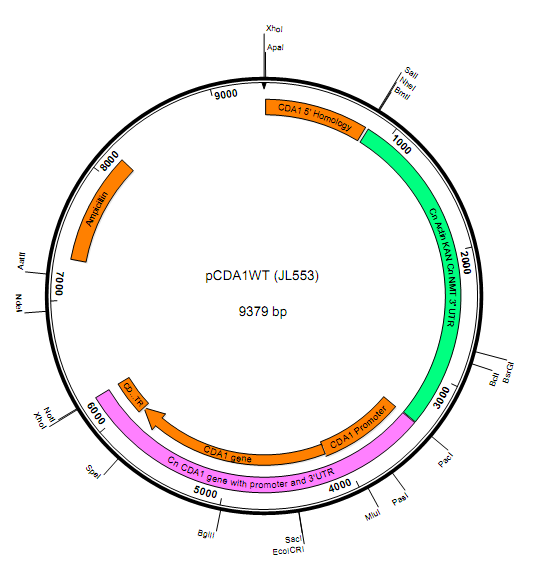


DNA sequence: pCDA1WT (JL553)

CTCGAGGGGCCCCGGTGACGTGGTGACAACGGGCACAGTGTGGCGCAATCCTCATCCTCCCGCAACATCCCCTCAGCTTGTTCTCTCATCCCAGCCCTATATCCATCATTGTTTTCTCATCTTCCCTGCACGATCCTGAACACCCATCTCATTCCTATCCCATTCTGCTCTCACAACACCACACTGCGCCCTCCCTGAAAAACCGCTTACCTTTCCCAAGTAGTGCGTAGCGGCCTCCTTGTTGTCAACACCCTCAATCTGGAGGAGAGACTGGTTAGGTCGGGAGTTTCGCTTTCCGCGCTTGTGCCCGAGGATTCGGCCCTTGATGTAGAGTCTATTAGACATTGCGTTGGCGGTTGGGAAGAAAGATTTAAGCAAGTCGTGAAAGTCGTTTGTGAAGGCGGAAATGGTCGATGGGGAAAATGATTCGAGCGGACAAATGCACCTGGTAAGCGGTGTCCTCTTGATCTGACAGCTTTCTACTCACCGGGAAGCCATCTTGTCTAGTCTTTTGAGGGATGGAGAAGTAGAACCAAACTTGTCAAACCCTTCCTTCAAAACGCGAACGTCCACCCTCAACGCATACGGCGAAACCCGGAGAATTGTTATGTGGTTGATTGAATCGCCGAATGTGGCACTTTGGTCGGCGATAAGAATGCGGTCGAGTCCGATGGTGTCGATACCCCCGGAAGGGAGCGACGCCGCCCCGCGACGCATGCATGAATGACCGACGAAAGATAACAAGTAATGCAGTACGAATTACTCGCTCCTCTTCTCTTCTGCGTGCATCTCTCTTGCGTTACATACATTGTACGGATAGCGTCGACGCTAGCCTGCGAGGATGTGAGCTGGAGAGCGGGGCACGGGAAGGGGAGGACTCACATAAGCATGCAGGATTCGAGTGGCATGGTGTGCGCTGAGTGTATGGTTGTCGGAGGAGAGGATGATGGTAACAACAATAGCAGCAACGTCACTCGACGCGCGTCCGGTGTGCCACACGGGGTAACGCCGAGTCGCCGTCAGGGTCGCCGAGACCACTCTCACAGCGTCACCGTTGGCACCAGCTCAGCTTACAGCTTCTATCCTCCGCCAGCATCCACATACATCCCCTATACCGCATCCCCCACCCACTGCCCAAGGTGAGTCATCTTCCCGCCCCCTTCCCTTGCCCGCCACTCAGTCCTCCATCCTCCACTAATCCACCTTATCGCACCCACCGCCTATCGCACATCCGAGCACAATGCTGGGCCTGCCAGGGGCTGCTAGATGGTGCTCTCCCCACGCTGATCTGCATGCCGGCCATTGGATCATGGGTGCTAGGTGCTGGGTGCTGGATGTTGGATGCTGGATGCTGGGTGCACGCTTGGTCATTTCCTTCCAGGATTGACGGTCGCCGAGAGGACGACGTGGCGTTCGACAACGAGGGCCGATAGCACCGCATCGCCTCGACCTGCATCCATCTGCCTTGTCCTTTTGGTGGAACAATCCATCCGTGCTGGTGCCACACGCATAGCTGGAAGAGATGGATGTGCGTTGAACAGAGCTGCCGTCAGGACTTTTTGGTGCACGGACCCTATTGTCCTCCCAATCTTCACCGCGTCTCCTAATATGCAGCCTCTTTGCTAATTGTCTTTTTCCATTAGTAAACTCGCCCGGATCCATGATTGAACAAGATGGATTGCACGCAGGTTCTCCGGCCGCTTGGGTGGAGAGGCTATTCGGCTATGACTGGGCACAACAGACAATCGGCTGCTCTGATGCCGCCGTGTTCCGGCTGTCAGCGCAGGGGCGCCCGGTTCTTTTTGTCAAGACCGACCTGTCCGGTGCCCTGAATGAACTGCAGGACGAGGCAGCGCGGCTATCGTGGCTGGCCACGACGGGCGTTCCTTGCGCAGCTGTGCTCGACGTTGTCACTGAAGCGGGAAGGGACTGGCTGCTATTGGGCGAAGTGCCGGGGCAGGATCTCCTGTCATCTCACCTTGCTCCTGCCGAGAAAGTATCCATCATGGCTGATGCAATGCGGCGGCTGCATACGCTTGATCCGGCTACCTGCCCATTCGACCACCAAGCGAAACATCGCATCGAGCGAGCACGTACTCGGATGGAAGCCGGTCTTGTCGATCAGGATGATCTGGACGAAGAGCATCAGGGGCTCGCGCCAGCCGAACTGTTCGCCAGGCTCAAGGCGCGCATGCCCGACGGCGAGGATCTCGTCGTGACCCATGGCGATGCCTGCTTGCCGAATATCATGGTGGAAAATGGCCGCTTTTCTGGATTCATCGACTGTGGCCGGCTGGGTGTGGCGGACCGCTATCAGGACATAGCGTTGGCTACCCGTGATATTGCTGAAGAGCTTGGCGGCGAATGGGCTGACCGCTTCCTCGTGCTTTACGGTATCGCCGCTCCCGATTCGCAGCGCATCGCCTTCTATCGCCTTCTTGACGAGTTCTTCTGAGAATTCCCCCTCAAACCCCGTCTCTTCATTCTCTCAATTTCTCTCGACCGCAACAAGCGGATAGACAGTCTTCTTGACATTCTTGATACATTGCTAACGCTGTGCTATGACATGCATATTTTGCCCAAGTACAAAAGTGTCACTCGACACTAATTGTAATTTCCCATTTACAACGGGAGACAAGAACCTGCAAACATGGACATACACAATGTAAAACATTCCAAGGAAAAAAAGGTAGAAGGAGTCTGATTTTACAGTCCTGTAGCTTGAATGATGTACACTAACCCAAGAATGAACACCGCCTCTGCCAACAAGAGTCTCCACTGTTTCCTCATCCAGCCGAGCATCTTGAACTGGGATTGATCACGCCCAAAATTCAAATCGCGTCAGCGCCCTTTATCCCGGAAACGACGATCAAAGCATTCGAATGGAAAATGACAGCAGATGGGAATCAGACTTACCGCGGGTACATCTGGTCCAAAAGGAAGCCCTTCAAAGGCAATTTCAAACCATGGCGTGGACGGCGATTCTAGCTCGTCTTCTTTATGAAGTCTCTTTTTCTTCTTCTTGGACGAGGAGGAGGCAGAGGAGCGGCGGTGTCTTATTTGGGGGGCCTTGTGTGCGTGCTTTCTTTTCTTTTGGATTTTTCGTCCGCGGGACGAGTTTGGTCGCTCTCTGTACCATGCTTGTGAGTTTGTTTATGCCTCCGTTTCCCCTTGCTTCTAGTCTCTTCCTTCTCCACAACTTTTCCATCTTCTTCTTCTTCTTCTCCTCCTTTTTCATCCTCATTTCCATCTACCTCTTCTTCCACTTTTTTTAAACTTTTTCCATCGCTATCCCTTTTGGGGTTTTCCCAACCGGTTCGGGACTCTCAAAACCCCAGAATCACCAGAGTCCTGCTTTCCTCCCTCTTCACCCTCTTGTCGTTAATTAATATCTGCATGACGTTCGGCTGCGAACGCTAGACTGTGAACTGTGACAACCCGATTCCGACATCAAAGTCTCGACGACACTTGTTGGATCGTCGAGGTTGAAAAGCATTATAGAAGACGTGCCCTGCCAAAAAGCGACAAAGTTACTTCCCAACAGGTCTATGATCTCATGAATTCTTGGCCGGCTTGAGCAGACACCACTTTCTGCCAACTTCTGTTTATTACCATGGATCGCGACGCGACGTGAAGAAAAGCAGCATCGGAACAATAACAAAGCACAACGCGACAAAAGCCGAACAGCGCAGGTCAGGGAGAGCATTTCTAGCGTGCCTTGGTAGATCGTTATCGCGATTTACTTTCCAGGCCCTGGGCGCTTCCAGCCATCAGCCAAAGGATAAAAGCGCGTGCCCTCTTCTTTTCATCATTAACTTTTATCCTCCTCAGCACCCACGCTCTGTGATTCCATCTCTTCCTCCTCGCATTCAAGCAGCCTCTTCATTTCTTTCCTCCGTCCCGGTGAGTGCGACGCCCGCCGCTGCCATTCCCACACGATGACTTGAGACGCGTCTTCCCGCTATAGCCGACGCCCCTTTTCGTTTTCTTGGCGTTTTGTCACATTGCCACATTGAGCAGCACAGCTTACTTGTCAGCAGCAAAAATCCAACTTCAAACAGCTCTTCAGCATCAACTCTATCACTCTTTCATCTCTTGTCAACTTCTCTTCCTTCTCGCTCCAAAAGCGGAATTTCGCCATGTTTACATTCGCTGCCTTCTCTGCTCTTCTAATTTCCCTCGCTGGTGTGGTGGCGCAGACTACAGGCACATCGGTTGACAGTAGCATCTTAACTAAGACTGCTGACTCTACCGGTCCCTCTGGTTTCTCCATTCCGTGAGTACTCTCGACTTTTCCGTCAACCTCCAGTCTCGCCACAGGCCATAGCGAACAATGAGCCAAGCGCCCACGCGAACCGTGCCCATCATTATCCTCCCACTAATTCCTTTAACCAAACGTAGCTTTTAGAGGCCAAATGCTGACAAGTGCGTTTTTAGTGCTTTGAGCGAGCTCACGTCTGGTGCCCCCACTGACTCTACTGTGGCCCTCTACTCTACCTTCGCGGCCGGTGCCACACCTACCGTTTCTGGTGCCCCTGTCCTCCCTACCAGTGCCCTCACCATCGCCGATTATCCAGCTTTAGATGTCACCCCTCCTACCAACTCCTCTTTGGTTAAGGACTGGATGGCCAAGGTGAGTTGTGTTTGAGTCCGAAAAGGCACCAGAAGAGCTAACAGTTGGATTAGATCGACTTGTCCAAGGTGCCCAGTTATAATGTGACAACGGGCGATTGTTCTACTGACGCGGCTGCTATCAGCGACGGTCGATGCTGGTGGACTTGTGGTGGTTGCACTCGGGAAACCGACATTGTCGAGTGTCCTGACAAGAATGTTTGGGGTCTCTCTTACGATGATGGGCCTTCTCCCTTCACCCCTCTCCTAATTGATTACCTTCAGGAGAAGAACATCAAGACCACCTTCTTCGTTGTCGGCTCTCGTGTCCTTTCTCGACCCGAGATGCTCCAAACCGAATACATGTCTGGACACCAGATCTCTATCCACACTTGGTCTCACCCCGCACTTACTACTCTTACCAACGAGGAAATTGTTGCCGAGCTTGGTTGGACAATGAAGGTCATCAAGGACACCCTTGGCGTCACCCCAAACACTTTCGCTCCCCCTTATGGTGACATTGATGACCGTGTTCGAGCTATTGCTGCTCAGATGGGCTTGACCCCTGTTATCTGGACTTCTTACACTGATGGCTCAACCACTGTTAACTTTGACACTGTAGGCTTATCTTGACTTTCGCAATAATCTTACTAACGAAATGACAGAACAACTGGCACATCAGTGGTGGTACCGCCACCGGCGCTTCTTCTTATGAGACCTTTGAGAAGATTCTCACCGAATACGCCCCAAAGTTGGACACTGGTTTCATCACTCTTGAGCACGACAGTAAGTCTTGTCTATCCGTCTTGCAATAATAATCCTGACGTATACCTTTACAGTCTACCAGCAGAGTGTTGACCTTGCTGTTGGTTACATTTTGCCCCAAGTTCTCGCCAACGGTACCTATCAGCTCAAATCCATCATCAACTGTTTGGGCAAGGACAGTAAGTTGCCTCCGCTAATCAGAAAAGGTTGTGGGCTAAGATGATACACAGCCTCCGAAGCATACATTGAGACTTCATCCAACCAGACTACTACTCAGATCACTGCAGCCACCGGCTCCCAGTCTACCTTCTTCCAGCCCATTGTTGGCACTGCTACCGGTGCTGAAGTCTCTGCACCTTCTGAGGCCACTGGCAGCACTGCCGCTGGCTCTGCTGCCTCCACCACTAGTGGTTCTGGCGCCAGCGCTTCTACAGGCGCCGCCTCTAACACTTCTTCCAGCGGGTCTGGTCGATCAGCCACCATGGGTGGTGCCCTCATTGCTCTTGCCGCTGTTGCGGTTGGTATGGTATATGTCGCCTAAGTATTTCAAGGCTTTCAATGTAACGATGGATGGGGATGGGTGGTGGGGGGGGAGGGAAGTGTGTCTAATGGGGCTATACTTGGGCTATACTTTGCCTCAAATCCATCAAGTATTAATAGCTGAACCATCTTTCGTTGAACCGTCTTTCATTGTGAACCATTTGTCTTTTTGATCTTTCAAAGTTTGATCCATTATGAATATCATGGACATTTTGAACGTTTTGAACATCCATGTACTTTTCATTCGATCGATCTGAACGTGTTGTTGTGCATACCTCGCGAACAAGCTTTCAATGGATGGCTCGAGGCGGCCGCCAATTCGCCCTATAGTGAGTCGTATTACAATTCACTGGCCGTCGTTTTACAACGTCGTGACTGGGAAAACCCTGGCGTTACCCAACTTAATCGCCTTGCAGCACATCCCCCTTTCGCCAGCTGGCGTAATAGCGAAGAGGCCCGCACCGATCGCCCTTCCCAACAGTTGCGCAGCCTGAATGGCGAATGGACGCGCCCTGTAGCGGCGCATTAAGCGCGGCGGGTGTGGTGGTTACGCGCAGCGTGACCGCTACACTTGCCAGCGCCCTAGCGCCCGCTCCTTTCGCTTTCTTCCCTTCCTTTCTCGCCACGTTCGCCGGCTTTCCCCGTCAAGCTCTAAATCGGGGGCTCCCTTTAGGGTTCCGATTTAGAGCTTTACGGCACCTCGACCGCAAAAAACTTGATTTGGGTGATGGTTCACGTAGTGGGCCATCGCCCTGATAGACGGTTTTTCGCCCTTTGACGTTGGAGTCCACGTTCTTTAATAGTGGACTCTTGTTCCAAACTGGAACAACACTCAACCCTATCTCGGTCTATTCTTTTGATTTATAAGGGATTTTGCCGATTTCGGCCTATTGGTTAAAAAATGAGCTGATTTAACAAATATTTAACGCGAATTTTAACAAAATATTAACGTTTACAATTTCGCCTGATGCGGTATTTTCTCCTTACGCATCTGTGCGGTATTTCACACCGCATATGGTGCACTCTCAGTACAATCTGCTCTGATGCCGCATAGTTAAGCCAGCCCCGACACCCGCCAACACCCGCTGACGCGCCCTGACGGGCTTGTCTGCTCCCGGCATCCGCTTACAGACAAGCTGTGACCGTCTCCGGGAGCTGCATGTGTCAGAGGTTTTCACCGTCATCACCGAAACGCGCGAGACGAAAGGGCCTCGTGATACGCCTATTTTTATAGGTTAATGTCATGATAATAATGGTTTCTTAGACGTCAGGTGGCACTTTTCGGGGAAATGTGCGCGGAACCCCTATTTGTTTATTTTTCTAAATACATTCAAATATGTATCCGCTCATGAGACAATAACCCTGATAAATGCTTCAATAATATTGAAAAAGGAAGAGTATGAGTATTCAACATTTCCGTGTCGCCCTTATTCCCTTTTTTGCGGCATTTTGCCTTCCTGTTTTTGCTCACCCAGAAACGCTGGTGAAAGTAAAAGATGCTGAAGATCAGTTGGGTGCACGAGTGGGTTACATCGAACTGGATCTCAACAGCGGTAAGATCCTTGAGAGTTTTCGCCCCGAAGAACGTTTTCCAATGATGAGCACTTTTAAAGTTCTGCTATGTGGCGCGGTATTATCCCGTATTGACGCCGGGCAAGAGCAACTCGGTCGCCGCATACACTATTCTCAGAATGACTTGGTTGAGTACTCACCAGTCACAGAAAAGCATCTTACGGATGGCATGACAGTAAGAGAATTATGCAGTGCTGCCATAACCATGAGTGATAACACTGCGGCCAACTTACTTCTGACAACGATCGGAGGACCGAAGGAGCTAACCGCTTTTTTGCACAACATGGGGGATCATGTAACTCGCCTTGATCGTTGGGAACCGGAGCTGAATGAAGCCATACCAAACGACGAGCGTGACACCACGATGCCTGTAGCAATGGCAACAACGTTGCGCAAACTATTAACTGGCGAACTACTTACTCTAGCTTCCCGGCAACAATTAATAGACTGGATGGAGGCGGATAAAGTTGCAGGACCACTTCTGCGCTCGGCCCTTCCGGCTGGCTGGTTTATTGCTGATAAATCTGGAGCCGGTGAGCGTGGGTCTCGCGGTATCATTGCAGCACTGGGGCCAGATGGTAAGCCCTCCCGTATCGTAGTTATCTACACGACGGGGAGTCAGGCAACTATGGATGAACGAAATAGACAGATCGCTGAGATAGGTGCCTCACTGATTAAGCATTGGTAACTGTCAGACCAAGTTTACTCATATATACTTTAGATTGATTTAAAACTTCATTTTTAATTTAAAAGGATCTAGGTGAAGATCCTTTTTGATAATCTCATGACCAAAATCCCTTAACGTGAGTTTTCGTTCCACTGAGCGTCAGACCCCGTAGAAAAGATCAAAGGATCTTCTTGAGATCCTTTTTTTCTGCGCGTAATCTGCTGCTTGCAAACAAAAAAACCACCGCTACCAGCGGTGGTTTGTTTGCCGGATCAAGAGCTACCAACTCTTTTTCCGAAGGTAACTGGCTTCAGCAGAGCGCAGATACCAAATACTGTCCTTCTAGTGTAGCCGTAGTTAGGCCACCACTTCAAGAACTCTGTAGCACCGCCTACATACCTCGCTCTGCTAATCCTGTTACCAGTGGCTGCTGCCAGTGGCGATAAGTCGTGTCTTACCGGGTTGGACTCAAGACGATAGTTACCGGATAAGGCGCAGCGGTCGGGCTGAACGGGGGGTTCGTGCACACAGCCCAGCTTGGAGCGAACGACCTACACCGAACTGAGATACCTACAGCGTGAGCTATGAGAAAGCGCCACGCTTCCCGAAGGGAGAAAGGCGGACAGGTATCCGGTAAGCGGCAGGGTCGGAACAGGAGAGCGCACGAGGGAGCTTCCAGGGGGAAACGCCTGGTATCTTTATAGTCCTGTCGGGTTTCGCCACCTCTGACTTGAGCGTCGATTTTTGTGATGCTCGTCAGGGGGGCGGAGCCTATGGAAAAACGCCAGCAACGCGGCCTTTTTACGGTTCCTGGCCTTTTGCTGGCCTTTTGCTCACATGTTCTTTCCTGCGTTATCCCCTGATTCTGTGGATAACCGTATTACCGCCTTTGAGTGAGCTGATACCGCTCGCCGCAGCCGAACGACCGAGCGCAGCGAGTCAGTGAGCGAGGAAGCGGAAGAGCGCCCAATACGCAAACCGCCTCTCCCCGCGCGTTGGCCGATTCATTAATGCAGCTGGCACGACAGGTTTCCCGACTGGAAAGCGGGCAGTGAGCGCAACGCAATTAATGTGAGTTAGCTCACTCATTAGGCACCCCAGGCTTTACACTTTATGCTTCCGGCTCGTATGTTGTGTGGAATTGTGAGCGGATAACAATTTCACACAGGAAACAGCTATGACCATGATTACGCCAAGCTATTTAGGTGACACTATAGAATACTC

The map and the sequences of pCDA1^CS^ (JL554) and pCDA1^MB^ (JL555) are similar to pCDA1WT except for the site specific mutations as indicated below.

Genomic sequence of wild-type and mutant version of the *C. neoformans* Cda1 gene (CNAG_05799). Sequences colored in grey are predicted introns obtained from fungidb.org.

Sequence of the Wild-type *CDA1*:

ATGTTTACATTCGCTGCCTTCTCTGCTCTTCTAATTTCCCTCGCTGGTGTGGTGGCGCAGACTACAGGCACATCGGTTGACAGTAGCATCTTAACTAAGACTGCTGACTCTACCGGTCCCTCTGGTTTCTCCATTCCGTGAGTACTCTCGACTTTTCCGTCAACCTCCAGTCTCGCCACAGGCCATAGCGAACAATGAGCCAAGCGCCCACGCGAACCGTGCCCATCATTATCCTCCCACTAATTCCTTTAACCAAACGTAGCTTTTAGAGGCCAAATGCTGACAAGTGCGTTTTTAGTGCTTTGAGCGAGCTCACGTCTGGTGCCCCCACTGACTCTACTGTGGCCCTCTACTCTACCTTCGCGGCCGGTGCCACACCTACCGTTTCTGGTGCCCCTGTCCTCCCTACCAGTGCCCTCACCATCGCCGATTATCCAGCTTTAGATGTCACCCCTCCTACCAACTCCTCTTTGGTTAAGGACTGGATGGCCAAGGTGAGTTGTGTTTGAGTCCGAAAAGGCACCAGAAGAGCTAACAGTTGGATTAGATCGACTTGTCCAAGGTGCCCAGTTATAATGTGACAACGGGCGATTGTTCTACTGACGCGGCTGCTATCAGCGACGGTCGATGCTGGTGGACTTGTGGTGGTTGCACTCGGGAAACCGACATTGTCGAGTGTCCTGACAAGAATGTTTGGGGTCTCTCTTACGATGATGGGCCTTCTCCCTTCACCCCTCTCCTAATTGATTACCTTCAGGAGAAGAACATCAAGACCACCTTCTTCGTTGTCGGCTCTCGTGTCCTTTCTCGACCCGAGATGCTCCAAACCGAATACATGTCTGGACACCAGATCTCTATCCACACTTGGTCTCACCCCGCACTTACTACTCTTACCAACGAGGAAATTGTTGCCGAGCTTGGTTGGACAATGAAGGTCATCAAGGACACCCTTGGCGTCACCCCAAACACTTTCCGACCCCCTTATGGTGACATTGATGACCGTGTTCGAGCTATTGCTGCTCAGATGGGCTTGACCCCTGTTATCTGGACTTCTTACACTGATGGCTCAACCACTGTTAACTTTGACACTGTAGGCTTATCTTGACTTTCGCAATAATCTTACTAACGAAATGACAGAACGACTGGCACATCAGTGGTGGTACCGCCACCGGCGCTTCTTCTTATGAGACCTTTGAGAAGATTCTCACCGAATACGCCCCAAAGTTGGACACTGGTTTCATCACTCTTGAGCACGACAGTAAGTCTTGTCTATCCGTCTTGCAATAATAATCCTGACGTATACCTTTACAGTCTACCAGCAGAGTGTTGACCTTGCTGTTGGTTACATTTTGCCCCAAGTTCTCGCCAACGGTACCTATCAGCTCAAATCCATCATCAACTGTTTGGGCAAGGACAGTAAGTTGCCTCCGCTAATCAGAAAAGGTTGTGGGCTAAGATGATACACAGCCTCCGAAGCATACATTGAGACTTCATCCAACCAGACTACTACTCAGATCACTGCAGCCACCGGCTCCCAGTCTACCTTCTTCCAGCCCATTGTTGGCACTGCTACCGGTGCTGAAGTCTCTGCACCTTCTGAGGCCACTGGCAGCACTGCCGCTGGCTCTGCTGCCTCCACCACTAGTGGTTCTGGCGCCAGCGCTTCTACAGGCGCCGCCTCTAACACTTCTTCCAGCGGGTCTGGTCGATCAGCCACCATGGGTGGTGCCCTCATTGCTCTTGCCGCTGTTGCGGTTGGTATGGTATATGTCGCCTAA.

Sequence of the catalytic mutant version of *CDA1*: (mutated residues are marked in red).

ATGTTTACATTCGCTGCCTTCTCTGCTCTTCTAATTTCCCTCGCTGGTGTGGTGGCGCAGACTACAGGCACATCGGTTGACAGTAGCATCTTAACTAAGACTGCTGACTCTACCGGTCCCTCTGGTTTCTCCATTCCGTGAGTACTCTCGACTTTTCCGTCAACCTCCAGTCTCGCCACAGGCCATAGCGAACAATGAGCCAAGCGCCCACGCGAACCGTGCCCATCATTATCCTCCCACTAATTCCTTTAACCAAACGTAGCTTTTAGAGGCCAAATGCTGACAAGTGCGTTTTTAGTGCTTTGAGCGAGCTCACGTCTGGTGCCCCCACTGACTCTACTGTGGCCCTCTACTCTACCTTCGCGGCCGGTGCCACACCTACCGTTTCTGGTGCCCCTGTCCTCCCTACCAGTGCCCTCACCATCGCCGATTATCCAGCTTTAGATGTCACCCCTCCTACCAACTCCTCTTTGGTTAAGGACTGGATGGCCAAGGTGAGTTGTGTTTGAGTCCGAAAAGGCACCAGAAGAGCTAACAGTTGGATTAGATCGACTTGTCCAAGGTGCCCAGTTATAATGTGACAACGGGCGATTGTTCTACTGACGCGGCTGCTATCAGCGACGGTCGATGCTGGTGGACTTGTGGTGGTTGCACTCGGGAAACCGACATTGTCGAGTGTCCTGACAAGAATGTTTGGGGTCTCTCTTACAACGATGGGCCTTCTCCCTTCACCCCTCTCCTAATTGATTACCTTCAGGAGAAGAACATCAAGACCACCTTCTTCGTTGTCGGCTCTCGTGTCCTTTCTCGACCCGAGATGCTCCAAACCGAATACATGTCTGGACACCAGATCTCTATCCACACTTGGTCTCACCCCGCACTTACTACTCTTACCAACGAGGAAATTGTTGCCGAGCTTGGTTGGACAATGAAGGTCATCAAGGACACCCTTGGCGTCACCCCAAACACTTTCGCTCCCCCTTATGGTGACATTGATGACCGTGTTCGAGCTATTGCTGCTCAGATGGGCTTGACCCCTGTTATCTGGACTTCTTACACTGATGGCTCAACCACTGTTAACTTTGACACTGTAGGCTTATCTTGACTTTCGCAATAATCTTACTAACGAAATGACAGAACAACTGGCACATCAGTGGTGGTACCGCCACCGGCGCTTCTTCTTATGAGACCTTTGAGAAGATTCTCACCGAATACGCCCCAAAGTTGGACACTGGTTTCATCACTCTTGAGCACGACAGTAAGTCTTGTCTATCCGTCTTGCAATAATAATCCTGACGTATACCTTTACAGTCTACCAGCAGAGTGTTGACCTTGCTGTTGGTTACATTTTGCCCCAAGTTCTCGCCAACGGTACCTATCAGCTCAAATCCATCATCAACTGTTTGGGCAAGGACAGTAAGTTGCCTCCGCTAATCAGAAAAGGTTGTGGGCTAAGATGATACACAGCCTCCGAAGCATACATTGAGACTTCATCCAACCAGACTACTACTCAGATCACTGCAGCCACCGGCTCCCAGTCTACCTTCTTCCAGCCCATTGTTGGCACTGCTACCGGTGCTGAAGTCTCTGCACCTTCTGAGGCCACTGGCAGCACTGCCGCTGGCTCTGCTGCCTCCACCACTAGTGGTTCTGGCGCCAGCGCTTCTACAGGCGCCGCCTCTAACACTTCTTCCAGCGGGTCTGGTCGATCAGCCACCATGGGTGGTGCCCTCATTGCTCTTGCCGCTGTTGCGGTTGGTATGGTATATGTCGCCTAA.

Sequence of the metal binding site mutant version of *CDA1*: (mutated residues are marked in red).

ATGTTTACATTCGCTGCCTTCTCTGCTCTTCTAATTTCCCTCGCTGGTGTGGTGGCGCAGACTACAGGCACATCGGTTGACAGTAGCATCTTAACTAAGACTGCTGACTCTACCGGTCCCTCTGGTTTCTCCATTCCGTGAGTACTCTCGACTTTTCCGTCAACCTCCAGTCTCGCCACAGGCCATAGCGAACAATGAGCCAAGCGCCCACGCGAACCGTGCCCATCATTATCCTCCCACTAATTCCTTTAACCAAACGTAGCTTTTAGAGGCCAAATGCTGACAAGTGCGTTTTTAGTGCTTTGAGCGAGCTCACGTCTGGTGCCCCCACTGACTCTACTGTGGCCCTCTACTCTACCTTCGCGGCCGGTGCCACACCTACCGTTTCTGGTGCCCCTGTCCTCCCTACCAGTGCCCTCACCATCGCCGATTATCCAGCTTTAGATGTCACCCCTCCTACCAACTCCTCTTTGGTTAAGGACTGGATGGCCAAGGTGAGTTGTGTTTGAGTCCGAAAAGGCACCAGAAGAGCTAACAGTTGGATTAGATCGACTTGTCCAAGGTGCCCAGTTATAATGTGACAACGGGCGATTGTTCTACTGACGCGGCTGCTATCAGCGACGGTCGATGCTGGTGGACTTGTGGTGGTTGCACTCGGGAAACCGACATTGTCGAGTGTCCTGACAAGAATGTTTGGGGTCTCTCTTACGATAACGGGCCTTCTCCCTTCACCCCTCTCCTAATTGATTACCTTCAGGAGAAGAACATCAAGACCACCTTCTTCGTTGTCGGCTCTCGTGTCCTTTCTCGACCCGAGATGCTCCAAACCGAATACATGTCTGGACACCAGATCTCTATCGCCACTTGGTCTGCCCCCGCACTTACTACTCTTACCAACGAGGAAATTGTTGCCGAGCTTGGTTGGACAATGAAGGTCATCAAGGACACCCTTGGCGTCACCCCAAACACTTTCGCTCCCCCTTATGGTGACATTGATGACCGTGTTCGAGCTATTGCTGCTCAGATGGGCTTGACCCCTGTTATCTGGACTTCTTACACTGATGGCTCAACCACTGTTAACTTTGACACTGTAGGCTTATCTTGACTTTCGCAATAATCTTACTAACGAAATGACAGAACAACTGGCACATCAGTGGTGGTACCGCCACCGGCGCTTCTTCTTATGAGACCTTTGAGAAGATTCTCACCGAATACGCCCCAAAGTTGGACACTGGTTTCATCACTCTTGAGCACGACAGTAAGTCTTGTCTATCCGTCTTGCAATAATAATCCTGACGTATACCTTTACAGTCTACCAGCAGAGTGTTGACCTTGCTGTTGGTTACATTTTGCCCCAAGTTCTCGCCAACGGTACCTATCAGCTCAAATCCATCATCAACTGTTTGGGCAAGGACAGTAAGTTGCCTCCGCTAATCAGAAAAGGTTGTGGGCTAAGATGATACACAGCCTCCGAAGCATACATTGAGACTTCATCCAACCAGACTACTACTCAGATCACTGCAGCCACCGGCTCCCAGTCTACCTTCTTCCAGCCCATTGTTGGCACTGCTACCGGTGCTGAAGTCTCTGCACCTTCTGAGGCCACTGGCAGCACTGCCGCTGGCTCTGCTGCCTCCACCACTAGTGGTTCTGGCGCCAGCGCTTCTACAGGCGCCGCCTCTAACACTTCTTCCAGCGGGTCTGGTCGATCAGCCACCATGGGTGGTGCCCTCATTGCTCTTGCCGCTGTTGCGGTTGGTATGGTATATGTCGCCTAA.

D

pCDA1WT (JL553)

CTCGAGGGGCCCCGGTGACGTGGTGACAACGGGCACAGTGTGGCGCAATCCTCATCCTCCCGCAACATCCCCTCAGCTTGTTCTCTCATCCCAGCCCTATATCCATCATTGTTTTCTCATCTTCCCTGCACGATCCTGAACACCCATCTCATTCCTATCCCATTCTGCTCTCACAACACCACACTGCGCCCTCCCTGAAAAACCGCTTACCTTTCCCAAGTAGTGCGTAGCGGCCTCCTTGTTGTCAACACCCTCAATCTGGAGGAGAGACTGGTTAGGTCGGGAGTTTCGCTTTCCGCGCTTGTGCCCGAGGATTCGGCCCTTGATGTAGAGTCTATTAGACATTGCGTTGGCGGTTGGGAAGAAAGATTTAAGCAAGTCGTGAAAGTCGTTTGTGAAGGCGGAAATGGTCGATGGGGAAAATGATTCGAGCGGACAAATGCACCTGGTAAGCGGTGTCCTCTTGATCTGACAGCTTTCTACTCACCGGGAAGCCATCTTGTCTAGTCTTTTGAGGGATGGAGAAGTAGAACCAAACTTGTCAAACCCTTCCTTCAAAACGCGAACGTCCACCCTCAACGCATACGGCGAAACCCGGAGAATTGTTATGTGGTTGATTGAATCGCCGAATGTGGCACTTTGGTCGGCGATAAGAATGCGGTCGAGTCCGATGGTGTCGATACCCCCGGAAGGGAGCGACGCCGCCCCGCGACGCATGCATGAATGACCGACGAAAGATAACAAGTAATGCAGTACGAATTACTCGCTCCTCTTCTCTTCTGCGTGCATCTCTCTTGCGTTACATACATTGTACGGATAGCGTCGACGCTAGCCTGCGAGGATGTGAGCTGGAGAGCGGGGCACGGGAAGGGGAGGACTCACATAAGCATGCAGGATTCGAGTGGCATGGTGTGCGCTGAGTGTATGGTTGTCGGAGGAGAGGATGATGGTAACAACAATAGCAGCAACGTCACTCGACGCGCGTCCGGTGTGCCACACGGGGTAACGCCGAGTCGCCGTCAGGGTCGCCGAGACCACTCTCACAGCGTCACCGTTGGCACCAGCTCAGCTTACAGCTTCTATCCTCCGCCAGCATCCACATACATCCCCTATACCGCATCCCCCACCCACTGCCCAAGGTGAGTCATCTTCCCGCCCCCTTCCCTTGCCCGCCACTCAGTCCTCCATCCTCCACTAATCCACCTTATCGCACCCACCGCCTATCGCACATCCGAGCACAATGCTGGGCCTGCCAGGGGCTGCTAGATGGTGCTCTCCCCACGCTGATCTGCATGCCGGCCATTGGATCATGGGTGCTAGGTGCTGGGTGCTGGATGTTGGATGCTGGATGCTGGGTGCACGCTTGGTCATTTCCTTCCAGGATTGACGGTCGCCGAGAGGACGACGTGGCGTTCGACAACGAGGGCCGATAGCACCGCATCGCCTCGACCTGCATCCATCTGCCTTGTCCTTTTGGTGGAACAATCCATCCGTGCTGGTGCCACACGCATAGCTGGAAGAGATGGATGTGCGTTGAACAGAGCTGCCGTCAGGACTTTTTGGTGCACGGACCCTATTGTCCTCCCAATCTTCACCGCGTCTCCTAATATGCAGCCTCTTTGCTAATTGTCTTTTTCCATTAGTAAACTCGCCCGGATCCATGATTGAACAAGATGGATTGCACGCAGGTTCTCCGGCCGCTTGGGTGGAGAGGCTATTCGGCTATGACTGGGCACAACAGACAATCGGCTGCTCTGATGCCGCCGTGTTCCGGCTGTCAGCGCAGGGGCGCCCGGTTCTTTTTGTCAAGACCGACCTGTCCGGTGCCCTGAATGAACTGCAGGACGAGGCAGCGCGGCTATCGTGGCTGGCCACGACGGGCGTTCCTTGCGCAGCTGTGCTCGACGTTGTCACTGAAGCGGGAAGGGACTGGCTGCTATTGGGCGAAGTGCCGGGGCAGGATCTCCTGTCATCTCACCTTGCTCCTGCCGAGAAAGTATCCATCATGGCTGATGCAATGCGGCGGCTGCATACGCTTGATCCGGCTACCTGCCCATTCGACCACCAAGCGAAACATCGCATCGAGCGAGCACGTACTCGGATGGAAGCCGGTCTTGTCGATCAGGATGATCTGGACGAAGAGCATCAGGGGCTCGCGCCAGCCGAACTGTTCGCCAGGCTCAAGGCGCGCATGCCCGACGGCGAGGATCTCGTCGTGACCCATGGCGATGCCTGCTTGCCGAATATCATGGTGGAAAATGGCCGCTTTTCTGGATTCATCGACTGTGGCCGGCTGGGTGTGGCGGACCGCTATCAGGACATAGCGTTGGCTACCCGTGATATTGCTGAAGAGCTTGGCGGCGAATGGGCTGACCGCTTCCTCGTGCTTTACGGTATCGCCGCTCCCGATTCGCAGCGCATCGCCTTCTATCGCCTTCTTGACGAGTTCTTCTGAGAATTCCCCCTCAAACCCCGTCTCTTCATTCTCTCAATTTCTCTCGACCGCAACAAGCGGATAGACAGTCTTCTTGACATTCTTGATACATTGCTAACGCTGTGCTATGACATGCATATTTTGCCCAAGTACAAAAGTGTCACTCGACACTAATTGTAATTTCCCATTTACAACGGGAGACAAGAACCTGCAAACATGGACATACACAATGTAAAACATTCCAAGGAAAAAAAGGTAGAAGGAGTCTGATTTTACAGTCCTGTAGCTTGAATGATGTACACTAACCCAAGAATGAACACCGCCTCTGCCAACAAGAGTCTCCACTGTTTCCTCATCCAGCCGAGCATCTTGAACTGGGATTGATCACGCCCAAAATTCAAATCGCGTCAGCGCCCTTTATCCCGGAAACGACGATCAAAGCATTCGAATGGAAAATGACAGCAGATGGGAATCAGACTTACCGCGGGTACATCTGGTCCAAAAGGAAGCCCTTCAAAGGCAATTTCAAACCATGGCGTGGACGGCGATTCTAGCTCGTCTTCTTTATGAAGTCTCTTTTTCTTCTTCTTGGACGAGGAGGAGGCAGAGGAGCGGCGGTGTCTTATTTGGGGGGCCTTGTGTGCGTGCTTTCTTTTCTTTTGGATTTTTCGTCCGCGGGACGAGTTTGGTCGCTCTCTGTACCATGCTTGTGAGTTTGTTTATGCCTCCGTTTCCCCTTGCTTCTAGTCTCTTCCTTCTCCACAACTTTTCCATCTTCTTCTTCTTCTTCTCCTCCTTTTTCATCCTCATTTCCATCTACCTCTTCTTCCACTTTTTTTAAACTTTTTCCATCGCTATCCCTTTTGGGGTTTTCCCAACCGGTTCGGGACTCTCAAAACCCCAGAATCACCAGAGTCCTGCTTTCCTCCCTCTTCACCCTCTTGTCGTTAATTAATATCTGCATGACGTTCGGCTGCGAACGCTAGACTGTGAACTGTGACAACCCGATTCCGACATCAAAGTCTCGACGACACTTGTTGGATCGTCGAGGTTGAAAAGCATTATAGAAGACGTGCCCTGCCAAAAAGCGACAAAGTTACTTCCCAACAGGTCTATGATCTCATGAATTCTTGGCCGGCTTGAGCAGACACCACTTTCTGCCAACTTCTGTTTATTACCATGGATCGCGACGCGACGTGAAGAAAAGCAGCATCGGAACAATAACAAAGCACAACGCGACAAAAGCCGAACAGCGCAGGTCAGGGAGAGCATTTCTAGCGTGCCTTGGTAGATCGTTATCGCGATTTACTTTCCAGGCCCTGGGCGCTTCCAGCCATCAGCCAAAGGATAAAAGCGCGTGCCCTCTTCTTTTCATCATTAACTTTTATCCTCCTCAGCACCCACGCTCTGTGATTCCATCTCTTCCTCCTCGCATTCAAGCAGCCTCTTCATTTCTTTCCTCCGTCCCGGTGAGTGCGACGCCCGCCGCTGCCATTCCCACACGATGACTTGAGACGCGTCTTCCCGCTATAGCCGACGCCCCTTTTCGTTTTCTTGGCGTTTTGTCACATTGCCACATTGAGCAGCACAGCTTACTTGTCAGCAGCAAAAATCCAACTTCAAACAGCTCTTCAGCATCAACTCTATCACTCTTTCATCTCTTGTCAACTTCTCTTCCTTCTCGCTCCAAAAGCGGAATTTCGCCATGTTTACATTCGCTGCCTTCTCTGCTCTTCTAATTTCCCTCGCTGGTGTGGTGGCGCAGACTACAGGCACATCGGTTGACAGTAGCATCTTAACTAAGACTGCTGACTCTACCGGTCCCTCTGGTTTCTCCATTCCGTGAGTACTCTCGACTTTTCCGTCAACCTCCAGTCTCGCCACAGGCCATAGCGAACAATGAGCCAAGCGCCCACGCGAACCGTGCCCATCATTATCCTCCCACTAATTCCTTTAACCAAACGTAGCTTTTAGAGGCCAAATGCTGACAAGTGCGTTTTTAGTGCTTTGAGCGAGCTCACGTCTGGTGCCCCCACTGACTCTACTGTGGCCCTCTACTCTACCTTCGCGGCCGGTGCCACACCTACCGTTTCTGGTGCCCCTGTCCTCCCTACCAGTGCCCTCACCATCGCCGATTATCCAGCTTTAGATGTCACCCCTCCTACCAACTCCTCTTTGGTTAAGGACTGGATGGCCAAGGTGAGTTGTGTTTGAGTCCGAAAAGGCACCAGAAGAGCTAACAGTTGGATTAGATCGACTTGTCCAAGGTGCCCAGTTATAATGTGACAACGGGCGATTGTTCTACTGACGCGGCTGCTATCAGCGACGGTCGATGCTGGTGGACTTGTGGTGGTTGCACTCGGGAAACCGACATTGTCGAGTGTCCTGACAAGAATGTTTGGGGTCTCTCTTACGATGATGGGCCTTCTCCCTTCACCCCTCTCCTAATTGATTACCTTCAGGAGAAGAACATCAAGACCACCTTCTTCGTTGTCGGCTCTCGTGTCCTTTCTCGACCCGAGATGCTCCAAACCGAATACATGTCTGGACACCAGATCTCTATCCACACTTGGTCTCACCCCGCACTTACTACTCTTACCAACGAGGAAATTGTTGCCGAGCTTGGTTGGACAATGAAGGTCATCAAGGACACCCTTGGCGTCACCCCAAACACTTTCGCTCCCCCTTATGGTGACATTGATGACCGTGTTCGAGCTATTGCTGCTCAGATGGGCTTGACCCCTGTTATCTGGACTTCTTACACTGATGGCTCAACCACTGTTAACTTTGACACTGTAGGCTTATCTTGACTTTCGCAATAATCTTACTAACGAAATGACAGAACAACTGGCACATCAGTGGTGGTACCGCCACCGGCGCTTCTTCTTATGAGACCTTTGAGAAGATTCTCACCGAATACGCCCCAAAGTTGGACACTGGTTTCATCACTCTTGAGCACGACAGTAAGTCTTGTCTATCCGTCTTGCAATAATAATCCTGACGTATACCTTTACAGTCTACCAGCAGAGTGTTGACCTTGCTGTTGGTTACATTTTGCCCCAAGTTCTCGCCAACGGTACCTATCAGCTCAAATCCATCATCAACTGTTTGGGCAAGGACAGTAAGTTGCCTCCGCTAATCAGAAAAGGTTGTGGGCTAAGATGATACACAGCCTCCGAAGCATACATTGAGACTTCATCCAACCAGACTACTACTCAGATCACTGCAGCCACCGGCTCCCAGTCTACCTTCTTCCAGCCCATTGTTGGCACTGCTACCGGTGCTGAAGTCTCTGCACCTTCTGAGGCCACTGGCAGCACTGCCGCTGGCTCTGCTGCCTCCACCACTAGTGGTTCTGGCGCCAGCGCTTCTACAGGCGCCGCCTCTAACACTTCTTCCAGCGGGTCTGGTCGATCAGCCACCATGGGTGGTGCCCTCATTGCTCTTGCCGCTGTTGCGGTTGGTATGGTATATGTCGCCTAAGTATTTCAAGGCTTTCAATGTAACGATGGATGGGGATGGGTGGTGGGGGGGGAGGGAAGTGTGTCTAATGGGGCTATACTTGGGCTATACTTTGCCTCAAATCCATCAAGTATTAATAGCTGAACCATCTTTCGTTGAACCGTCTTTCATTGTGAACCATTTGTCTTTTTGATCTTTCAAAGTTTGATCCATTATGAATATCATGGACATTTTGAACGTTTTGAACATCCATGTACTTTTCATTCGATCGATCTGAACGTGTTGTTGTGCATACCTCGCGAACAAGCTTTCAATGGATGGCTCGAGGCGGCCGCCAATTCGCCCTATAGTGAGTCGTATTACAATTCACTGGCCGTCGTTTTACAACGTCGTGACTGGGAAAACCCTGGCGTTACCCAACTTAATCGCCTTGCAGCACATCCCCCTTTCGCCAGCTGGCGTAATAGCGAAGAGGCCCGCACCGATCGCCCTTCCCAACAGTTGCGCAGCCTGAATGGCGAATGGACGCGCCCTGTAGCGGCGCATTAAGCGCGGCGGGTGTGGTGGTTACGCGCAGCGTGACCGCTACACTTGCCAGCGCCCTAGCGCCCGCTCCTTTCGCTTTCTTCCCTTCCTTTCTCGCCACGTTCGCCGGCTTTCCCCGTCAAGCTCTAAATCGGGGGCTCCCTTTAGGGTTCCGATTTAGAGCTTTACGGCACCTCGACCGCAAAAAACTTGATTTGGGTGATGGTTCACGTAGTGGGCCATCGCCCTGATAGACGGTTTTTCGCCCTTTGACGTTGGAGTCCACGTTCTTTAATAGTGGACTCTTGTTCCAAACTGGAACAACACTCAACCCTATCTCGGTCTATTCTTTTGATTTATAAGGGATTTTGCCGATTTCGGCCTATTGGTTAAAAAATGAGCTGATTTAACAAATATTTAACGCGAATTTTAACAAAATATTAACGTTTACAATTTCGCCTGATGCGGTATTTTCTCCTTACGCATCTGTGCGGTATTTCACACCGCATATGGTGCACTCTCAGTACAATCTGCTCTGATGCCGCATAGTTAAGCCAGCCCCGACACCCGCCAACACCCGCTGACGCGCCCTGACGGGCTTGTCTGCTCCCGGCATCCGCTTACAGACAAGCTGTGACCGTCTCCGGGAGCTGCATGTGTCAGAGGTTTTCACCGTCATCACCGAAACGCGCGAGACGAAAGGGCCTCGTGATACGCCTATTTTTATAGGTTAATGTCATGATAATAATGGTTTCTTAGACGTCAGGTGGCACTTTTCGGGGAAATGTGCGCGGAACCCCTATTTGTTTATTTTTCTAAATACATTCAAATATGTATCCGCTCATGAGACAATAACCCTGATAAATGCTTCAATAATATTGAAAAAGGAAGAGTATGAGTATTCAACATTTCCGTGTCGCCCTTATTCCCTTTTTTGCGGCATTTTGCCTTCCTGTTTTTGCTCACCCAGAAACGCTGGTGAAAGTAAAAGATGCTGAAGATCAGTTGGGTGCACGAGTGGGTTACATCGAACTGGATCTCAACAGCGGTAAGATCCTTGAGAGTTTTCGCCCCGAAGAACGTTTTCCAATGATGAGCACTTTTAAAGTTCTGCTATGTGGCGCGGTATTATCCCGTATTGACGCCGGGCAAGAGCAACTCGGTCGCCGCATACACTATTCTCAGAATGACTTGGTTGAGTACTCACCAGTCACAGAAAAGCATCTTACGGATGGCATGACAGTAAGAGAATTATGCAGTGCTGCCATAACCATGAGTGATAACACTGCGGCCAACTTACTTCTGACAACGATCGGAGGACCGAAGGAGCTAACCGCTTTTTTGCACAACATGGGGGATCATGTAACTCGCCTTGATCGTTGGGAACCGGAGCTGAATGAAGCCATACCAAACGACGAGCGTGACACCACGATGCCTGTAGCAATGGCAACAACGTTGCGCAAACTATTAACTGGCGAACTACTTACTCTAGCTTCCCGGCAACAATTAATAGACTGGATGGAGGCGGATAAAGTTGCAGGACCACTTCTGCGCTCGGCCCTTCCGGCTGGCTGGTTTATTGCTGATAAATCTGGAGCCGGTGAGCGTGGGTCTCGCGGTATCATTGCAGCACTGGGGCCAGATGGTAAGCCCTCCCGTATCGTAGTTATCTACACGACGGGGAGTCAGGCAACTATGGATGAACGAAATAGACAGATCGCTGAGATAGGTGCCTCACTGATTAAGCATTGGTAACTGTCAGACCAAGTTTACTCATATATACTTTAGATTGATTTAAAACTTCATTTTTAATTTAAAAGGATCTAGGTGAAGATCCTTTTTGATAATCTCATGACCAAAATCCCTTAACGTGAGTTTTCGTTCCACTGAGCGTCAGACCCCGTAGAAAAGATCAAAGGATCTTCTTGAGATCCTTTTTTTCTGCGCGTAATCTGCTGCTTGCAAACAAAAAAACCACCGCTACCAGCGGTGGTTTGTTTGCCGGATCAAGAGCTACCAACTCTTTTTCCGAAGGTAACTGGCTTCAGCAGAGCGCAGATACCAAATACTGTCCTTCTAGTGTAGCCGTAGTTAGGCCACCACTTCAAGAACTCTGTAGCACCGCCTACATACCTCGCTCTGCTAATCCTGTTACCAGTGGCTGCTGCCAGTGGCGATAAGTCGTGTCTTACCGGGTTGGACTCAAGACGATAGTTACCGGATAAGGCGCAGCGGTCGGGCTGAACGGGGGGTTCGTGCACACAGCCCAGCTTGGAGCGAACGACCTACACCGAACTGAGATACCTACAGCGTGAGCTATGAGAAAGCGCCACGCTTCCCGAAGGGAGAAAGGCGGACAGGTATCCGGTAAGCGGCAGGGTCGGAACAGGAGAGCGCACGAGGGAGCTTCCAGGGGGAAACGCCTGGTATCTTTATAGTCCTGTCGGGTTTCGCCACCTCTGACTTGAGCGTCGATTTTTGTGATGCTCGTCAGGGGGGCGGAGCCTATGGAAAAACGCCAGCAACGCGGCCTTTTTACGGTTCCTGGCCTTTTGCTGGCCTTTTGCTCACATGTTCTTTCCTGCGTTATCCCCTGATTCTGTGGATAACCGTATTACCGCCTTTGAGTGAGCTGATACCGCTCGCCGCAGCCGAACGACCGAGCGCAGCGAGTCAGTGAGCGAGGAAGCGGAAGAGCGCCCAATACGCAAACCGCCTCTCCCCGCGCGTTGGCCGATTCATTAATGCAGCTGGCACGACAGGTTTCCCGACTGGAAAGCGGGCAGTGAGCGCAACGCAATTAATGTGAGTTAGCTCACTCATTAGGCACCCCAGGCTTTACACTTTATGCTTCCGGCTCGTATGTTGTGTGGAATTGTGAGCGGATAACAATTTCACACAGGAAACAGCTATGACCATGATTACGCCAAGCTATTTAGGTGACACTATAGAATACTC

Wild-type *CDA1*:

ATGTTTACATTCGCTGCCTTCTCTGCTCTTCTAATTTCCCTCGCTGGTGTGGTGGCGCAGACTACAGGCACATCGGTTGACAGTAGCATCTTAACTAAGACTGCTGACTCTACCGGTCCCTCTGGTTTCTCCATTCCGTGAGTACTCTCGACTTTTCCGTCAACCTCCAGTCTCGCCACAGGCCATAGCGAACAATGAGCCAAGCGCCCACGCGAACCGTGCCCATCATTATCCTCCCACTAATTCCTTTAACCAAACGTAGCTTTTAGAGGCCAAATGCTGACAAGTGCGTTTTTAGTGCTTTGAGCGAGCTCACGTCTGGTGCCCCCACTGACTCTACTGTGGCCCTCTACTCTACCTTCGCGGCCGGTGCCACACCTACCGTTTCTGGTGCCCCTGTCCTCCCTACCAGTGCCCTCACCATCGCCGATTATCCAGCTTTAGATGTCACCCCTCCTACCAACTCCTCTTTGGTTAAGGACTGGATGGCCAAGGTGAGTTGTGTTTGAGTCCGAAAAGGCACCAGAAGAGCTAACAGTTGGATTAGATCGACTTGTCCAAGGTGCCCAGTTATAATGTGACAACGGGCGATTGTTCTACTGACGCGGCTGCTATCAGCGACGGTCGATGCTGGTGGACTTGTGGTGGTTGCACTCGGGAAACCGACATTGTCGAGTGTCCTGACAAGAATGTTTGGGGTCTCTCTTACGATGATGGGCCTTCTCCCTTCACCCCTCTCCTAATTGATTACCTTCAGGAGAAGAACATCAAGACCACCTTCTTCGTTGTCGGCTCTCGTGTCCTTTCTCGACCCGAGATGCTCCAAACCGAATACATGTCTGGACACCAGATCTCTATCCACACTTGGTCTCACCCCGCACTTACTACTCTTACCAACGAGGAAATTGTTGCCGAGCTTGGTTGGACAATGAAGGTCATCAAGGACACCCTTGGCGTCACCCCAAACACTTTCCGACCCCCTTATGGTGACATTGATGACCGTGTTCGAGCTATTGCTGCTCAGATGGGCTTGACCCCTGTTATCTGGACTTCTTACACTGATGGCTCAACCACTGTTAACTTTGACACTGTAGGCTTATCTTGACTTTCGCAATAATCTTACTAACGAAATGACAGAACGACTGGCACATCAGTGGTGGTACCGCCACCGGCGCTTCTTCTTATGAGACCTTTGAGAAGATTCTCACCGAATACGCCCCAAAGTTGGACACTGGTTTCATCACTCTTGAGCACGACAGTAAGTCTTGTCTATCCGTCTTGCAATAATAATCCTGACGTATACCTTTACAGTCTACCAGCAGAGTGTTGACCTTGCTGTTGGTTACATTTTGCCCCAAGTTCTCGCCAACGGTACCTATCAGCTCAAATCCATCATCAACTGTTTGGGCAAGGACAGTAAGTTGCCTCCGCTAATCAGAAAAGGTTGTGGGCTAAGATGATACACAGCCTCCGAAGCATACATTGAGACTTCATCCAACCAGACTACTACTCAGATCACTGCAGCCACCGGCTCCCAGTCTACCTTCTTCCAGCCCATTGTTGGCACTGCTACCGGTGCTGAAGTCTCTGCACCTTCTGAGGCCACTGGCAGCACTGCCGCTGGCTCTGCTGCCTCCACCACTAGTGGTTCTGGCGCCAGCGCTTCTACAGGCGCCGCCTCTAACACTTCTTCCAGCGGGTCTGGTCGATCAGCCACCATGGGTGGTGCCCTCATTGCTCTTGCCGCTGTTGCGGTTGGTATGGTATATGTCGCCTAA.

*cda1^CS^*: (mutated residues are marked in red).

ATGTTTACATTCGCTGCCTTCTCTGCTCTTCTAATTTCCCTCGCTGGTGTGGTGGCGCAGACTACAGGCACATCGGTTGACAGTAGCATCTTAACTAAGACTGCTGACTCTACCGGTCCCTCTGGTTTCTCCATTCCGTGAGTACTCTCGACTTTTCCGTCAACCTCCAGTCTCGCCACAGGCCATAGCGAACAATGAGCCAAGCGCCCACGCGAACCGTGCCCATCATTATCCTCCCACTAATTCCTTTAACCAAACGTAGCTTTTAGAGGCCAAATGCTGACAAGTGCGTTTTTAGTGCTTTGAGCGAGCTCACGTCTGGTGCCCCCACTGACTCTACTGTGGCCCTCTACTCTACCTTCGCGGCCGGTGCCACACCTACCGTTTCTGGTGCCCCTGTCCTCCCTACCAGTGCCCTCACCATCGCCGATTATCCAGCTTTAGATGTCACCCCTCCTACCAACTCCTCTTTGGTTAAGGACTGGATGGCCAAGGTGAGTTGTGTTTGAGTCCGAAAAGGCACCAGAAGAGCTAACAGTTGGATTAGATCGACTTGTCCAAGGTGCCCAGTTATAATGTGACAACGGGCGATTGTTCTACTGACGCGGCTGCTATCAGCGACGGTCGATGCTGGTGGACTTGTGGTGGTTGCACTCGGGAAACCGACATTGTCGAGTGTCCTGACAAGAATGTTTGGGGTCTCTCTTAC**A**A**C**GATGGGCCTTCTCCCTTCACCCCTCTCCTAATTGATTACCTTCAGGAGAAGAACATCAAGACCACCTTCTTCGTTGTCGGCTCTCGTGTCCTTTCTCGACCCGAGATGCTCCAAACCGAATACATGTCTGGACACCAGATCTCTATCCACACTTGGTCTCACCCCGCACTTACTACTCTTACCAACGAGGAAATTGTTGCCGAGCTTGGTTGGACAATGAAGGTCATCAAGGACACCCTTGGCGTCACCCCAAACACTTTC**GCT**CCCCCTTATGGTGACATTGATGACCGTGTTCGAGCTATTGCTGCTCAGATGGGCTTGACCCCTGTTATCTGGACTTCTTACACTGATGGCTCAACCACTGTTAACTTTGACACTGTAGGCTTATCTTGACTTTCGCAATAATCTTACTAACGAAATGACAGAAC**A**ACTGGCACATCAGTGGTGGTACCGCCACCGGCGCTTCTTCTTATGAGACCTTTGAGAAGATTCTCACCGAATACGCCCCAAAGTTGGACACTGGTTTCATCACTCTTGAGCACGACAGTAAGTCTTGTCTATCCGTCTTGCAATAATAATCCTGACGTATACCTTTACAGTCTACCAGCAGAGTGTTGACCTTGCTGTTGGTTACATTTTGCCCCAAGTTCTCGCCAACGGTACCTATCAGCTCAAATCCATCATCAACTGTTTGGGCAAGGACAGTAAGTTGCCTCCGCTAATCAGAAAAGGTTGTGGGCTAAGATGATACACAGCCTCCGAAGCATACATTGAGACTTCATCCAACCAGACTACTACTCAGATCACTGCAGCCACCGGCTCCCAGTCTACCTTCTTCCAGCCCATTGTTGGCACTGCTACCGGTGCTGAAGTCTCTGCACCTTCTGAGGCCACTGGCAGCACTGCCGCTGGCTCTGCTGCCTCCACCACTAGTGGTTCTGGCGCCAGCGCTTCTACAGGCGCCGCCTCTAACACTTCTTCCAGCGGGTCTGGTCGATCAGCCACCATGGGTGGTGCCCTCATTGCTCTTGCCGCTGTTGCGGTTGGTATGGTATATGTCGCCTAA.

*cda1^MB^*: (mutated residues are marked in red).

ATGTTTACATTCGCTGCCTTCTCTGCTCTTCTAATTTCCCTCGCTGGTGTGGTGGCGCAGACTACAGGCACATCGGTTGACAGTAGCATCTTAACTAAGACTGCTGACTCTACCGGTCCCTCTGGTTTCTCCATTCCGTGAGTACTCTCGACTTTTCCGTCAACCTCCAGTCTCGCCACAGGCCATAGCGAACAATGAGCCAAGCGCCCACGCGAACCGTGCCCATCATTATCCTCCCACTAATTCCTTTAACCAAACGTAGCTTTTAGAGGCCAAATGCTGACAAGTGCGTTTTTAGTGCTTTGAGCGAGCTCACGTCTGGTGCCCCCACTGACTCTACTGTGGCCCTCTACTCTACCTTCGCGGCCGGTGCCACACCTACCGTTTCTGGTGCCCCTGTCCTCCCTACCAGTGCCCTCACCATCGCCGATTATCCAGCTTTAGATGTCACCCCTCCTACCAACTCCTCTTTGGTTAAGGACTGGATGGCCAAGGTGAGTTGTGTTTGAGTCCGAAAAGGCACCAGAAGAGCTAACAGTTGGATTAGATCGACTTGTCCAAGGTGCCCAGTTATAATGTGACAACGGGCGATTGTTCTACTGACGCGGCTGCTATCAGCGACGGTCGATGCTGGTGGACTTGTGGTGGTTGCACTCGGGAAACCGACATTGTCGAGTGTCCTGACAAGAATGTTTGGGGTCTCTCTTACGAT**A**A**C**GGGCCTTCTCCCTTCACCCCTCTCCTAATTGATTACCTTCAGGAGAAGAACATCAAGACCACCTTCTTCGTTGTCGGCTCTCGTGTCCTTTCTCGACCCGAGATGCTCCAAACCGAATACATGTCTGGACACCAGATCTCTATC**GC**CACTTGGTCT**GC**CCCCGCACTTACTACTCTTACCAACGAGGAAATTGTTGCCGAGCTTGGTTGGACAATGAAGGTCATCAAGGACACCCTTGGCGTCACCCCAAACACTTTCGCTCCCCCTTATGGTGACATTGATGACCGTGTTCGAGCTATTGCTGCTCAGATGGGCTTGACCCCTGTTATCTGGACTTCTTACACTGATGGCTCAACCACTGTTAACTTTGACACTGTAGGCTTATCTTGACTTTCGCAATAATCTTACTAACGAAATGACAGAACAACTGGCACATCAGTGGTGGTACCGCCACCGGCGCTTCTTCTTATGAGACCTTTGAGAAGATTCTCACCGAATACGCCCCAAAGTTGGACACTGGTTTCATCACTCTTGAGCACGACAGTAAGTCTTGTCTATCCGTCTTGCAATAATAATCCTGACGTATACCTTTACAGTCTACCAGCAGAGTGTTGACCTTGCTGTTGGTTACATTTTGCCCCAAGTTCTCGCCAACGGTACCTATCAGCTCAAATCCATCATCAACTGTTTGGGCAAGGACAGTAAGTTGCCTCCGCTAATCAGAAAAGGTTGTGGGCTAAGATGATACACAGCCTCCGAAGCATACATTGAGACTTCATCCAACCAGACTACTACTCAGATCACTGCAGCCACCGGCTCCCAGTCTACCTTCTTCCAGCCCATTGTTGGCACTGCTACCGGTGCTGAAGTCTCTGCACCTTCTGAGGCCACTGGCAGCACTGCCGCTGGCTCTGCTGCCTCCACCACTAGTGGTTCTGGCGCCAGCGCTTCTACAGGCGCCGCCTCTAACACTTCTTCCAGCGGGTCTGGTCGATCAGCCACCATGGGTGGTGCCCTCATTGCTCTTGCCGCTGTTGCGGTTGGTATGGTATATGTCGCCTAA.
