# Supplementary material for: Cryptococcus neoformans Cda1 and Its Chitin Deacetylase Activity Are Required for Fungal Pathogenesis
Source: mBio. 2018 Nov 20;9(6):e02087-18. doi: 10.1128/mBio.02087-18 (PMC6247093; doi:10.1128/mBio.02087-18)
Supplement: FIG S2 [file mbo006184181sf2.docx]

Fig. S2:

L

C

M

CW

L

C

M

CW

L

C

M

CW

L

C

M

CW

L

C

M

CW

KN99

*cda1Δ*

*cda1*^CS^:A

*cda1*^CS^:B

*cda1Δ::CDA1*
