# Supplementary material for: Cryptococcus neoformans Cda1 and Its Chitin Deacetylase Activity Are Required for Fungal Pathogenesis
Source: mBio. 2018 Nov 20;9(6):e02087-18. doi: 10.1128/mBio.02087-18 (PMC6247093; doi:10.1128/mBio.02087-18)
Supplement: TABLE S1 [file mbo006184181st1.docx]

Table S1: Primers used in this study.

| Generation of *CDA* wild-type and mutant fragments | |
| --- | --- |
| CDA-1 | CTCGAGGGGCCCCGGTGACGTGGTGACAACGGGCAC |
| CDA-2 | GCTAGCGTCGACGCTATCCGTACAATGTATGTAACGCA |
| CDA-3 | GTCCTGACAAGAATGTTTGGGGTCTC |
| CDA-4 | TGGCGGTACCACCACTGATGTGC |
| CDA-5 | CCGGCGCTTCTTCTTATGAGACC |
| CDA-6 | CTCGAGCCATCCATTGAAAGCTTGTTCGCG |
| Generation of CDA15’ homology +G418 fragment | |
| CDA15’-1 | CTCGAGGGGCCCCGGTGACGTGGTGACAACGGGCAC |
| CDA15’-2 | GCTAGCGTCGACGCTATCCGTACAATGTATGTAACGCA |
| G418-1 | CTGCGAGGATGTGAGCTGGAGAG |
| G418-2 | TTAATTAACGACAAGAGGGTGAAGAGGGAGGAA |
| Primers used for the diagnostic PCR screening of the transformants | |
| CDADIA-1 | TGGTTCGGAACTTGGACTTGACAGC |
| CDADIA-2 | AGGCATTCTGCTGCATTTATGTGC |
| CDADIA-3 | ATAGAAGCTGTAAGCTGAGCTGGTGC |
| Primers used for the real time quantitative PCR | |
| CDA1RTF | cttcttacactgatggctcaac |
| CDA1RTR | caacactctgctggtagatgtc |
| CDA2RTF | GCTGTCAGTACAGACATCAACACC |
| CDA2RTR | TACATCATTTGGAATCTTGGTATTAAG |
| CDA3RTF | GCGTAGAGGACGATCTGTACTCTC |
| CDA3RTR | ACTGACTGTGGGGAAGTAATAACATT |
| Primers used for the sequencing of CDA1 gene | |
| CDA1SeqFor1 | AACTTCAAACAGCTCTTCAGC |
| CDA1SeqFor2 | GTGGTTGCACTCGGGAAACC |
| CDA1SeqFor3 | ATCTACCAGCAGAGTGTTGACC |
